# *Leptotrichia* species in human infections II

**DOI:** 10.1080/20002297.2017.1368848

**Published:** 2017-09-15

**Authors:** Emenike R. K. Eribe, Ingar Olsen

**Affiliations:** ^a^ Department of Oral Biology, Faculty of Dentistry, University of Oslo, Oslo, Norway

**Keywords:** *L*eptotrichia species, taxonomy, opportunistic pathogens, Crispr-Cas, CSIs

## Abstract

*Leptotrichia* species are non-motile facultative anaerobic/anaerobic bacteria that are found mostly in the oral cavity and some other parts of the human body, in animals, and even in ocean sediments. Valid species include *L. buccalis*, *L. goodfellowii*, *L. hofstadii*, *L. honkongensis*, *L. shahii*, *L. trevisanii*, and *L. wadei*. Some species require serum or blood for growth. All species ferment carbohydrates and produce lactic acid that may be involved with tooth decay. Acting as opportunistic pathogens, they are involved in a variety of diseases, and have been isolated from immunocompromised but also immunocompetent individuals. Mucositis, oral lesions, wounds, and abscesses may predispose to *Leptotrichia* septicemia. Because identification of *Leptotrichia* species by phenotypic features occasionally lead to misidentification, genetic techniques such as 16S rRNA gene sequencing is recommended. Early diagnosis and treatment of leptotrichia infections is important for positive outcomes. Over the last years, *Leptotrichia* species have been associated with several changes in taxonomy and new associations with clinical diseases. Such changes are reported in this updated review.

## Introduction

**Figure 1.**
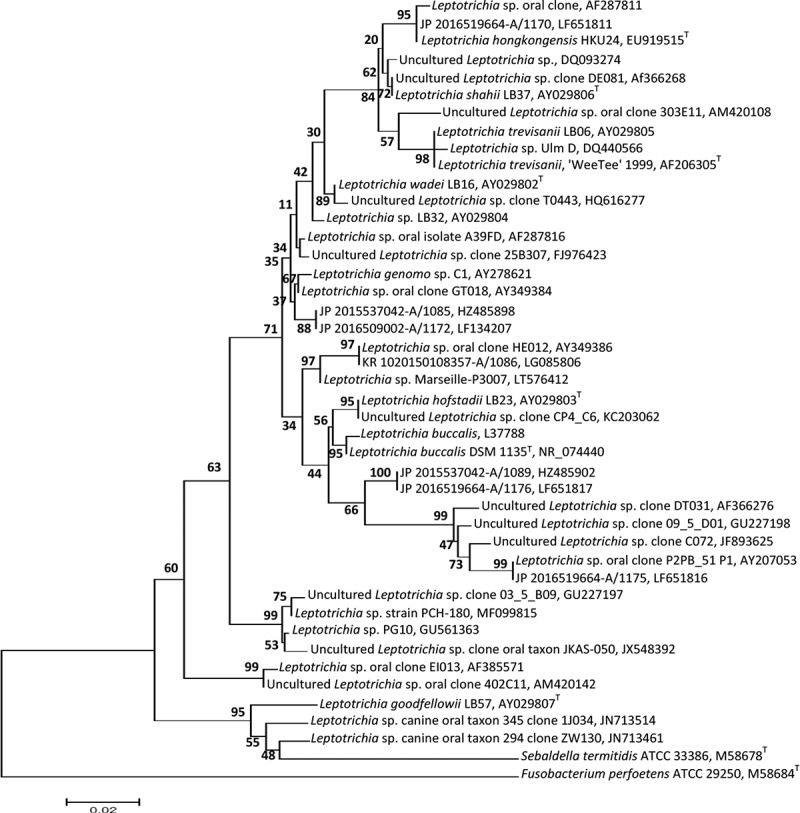
A phylogenetic tree obtained from the MEGA (www.megasoftware.net) program based on only sequences >800 bp by neighbor joining after ClustalW alignment. The analysis of the 16S rRNA gene sequences of the representative clones and reference strains of related *Leptotrichia* species and other members of *Fusobacteriacea* derived from GenBank is shown. Bootstrap values from 500 replicate trees are given at the nodes. Scale bar shows sequence divergence. ^T^ = type strain.

*Leptotrichia* is one of four genera within the family *Leptotrichiaceae*. Description of *Leptotrichiaceae* is based on phylogenetic analyses of the 16S rRNA gene sequences. *Leptotrichia* species are facultative anaerobic/anaerobic Gram-negative rods that inhabit the oral cavity, intestines, urogenital system, and female genital tract of humans [1–5]. They are non-motile and ferment carbohydrates to produce various organic acids, including lactic acid, and traces of acetic, formic, or succinic acid, depending on the substrates and species. Some species are fastidious, requiring serum or blood for growth [1–3]. *L*. *buccalis* was for centuries the only known *Leptotrichia* species, but new species have now been formally accepted, which include *L. goodfellowii*, *L. hofstadii*, *L. shahii*, *L. trevisanii*, and *L. wadei* (Figure 1) [2,4,5] and *L. hongkongensis* [6]. As with other members of the oral commensal microbiota, *Leptotrichia* species are also associated with periodontal diseases and oral cavity abscesses [5,7,8], typically as opportunistic infections. However, isolation of *Leptotrichia* species from infective endocarditis patients with normally functioning immune systems has been also reported [5,9–12]. *Leptotrichia* species can cause opportunistic infections that lead to bacteremia in neutropenic patients with oral mucosal injuries [2,5] and bacteremia due to *L. trevisanii* after an allogeneic bone-marrow transplant [13]. Although systemic infections involving *Leptotrichia* species are infrequent, severe infections have been reported in immunocompromised patients [2,4,7,9,10,13–19].

Some species have been recovered from the human oral cavity, while others such as *L. buccalis* and *L. goodfellowii* have been recovered from dog bites [20] and guinea-pig wounds [6,21]. Based on 16S rDNAsequences comparisons *Leptotrichia* species were isolated from the hindgut of termites, fish, and even ocean sediments (Table 2) [3]. Most mammals may have their own versions of human oral species, which are typically host-species specific.

**Table 1.** *Leptotrichia* completed genome assembly sequences

	Species	Short description of species	Median total length (Mb)	Median gene count	Median protein count	Median GC%	Accession number	Depositor or source
1	*Leptotrichia buccalis*	This Gram-negative rod is a member of the normal human oral microbial community but has occasionally been implicated in cases of septicemia and endocarditis	2.46561	2,309	2,182	29.6	NC_013192	JGI-PGF
2	*Leptotrichia goodfellowii*	The species contain Gram-negative anaerobic rods isolated from human sources (blood)	2.28422	2,199	2,079	31.55	AZXW00000000	JGI-PGF
3	*Leptotrichia hofstadii*	Gram-negative, non-spore-forming, non-motile rods isolated from the saliva of a healthy person	2.50859	2,413	2,156	30.65	AUAY00000000	JGI-PGF
4	*Leptotrichia shahii*	Gram-negative, non-spore-forming, non-motile rods isolated from a gingivitis patient	2.14461	1,982	1,888	29.5	ARDD00000000	JGI-PGF
5	*Leptotrichia trevisanii*	Gram-negative, aerobic, non-spore-forming, non-motile rods, isolated from blood of a patient with acute myeloid leukemia	2.85336	2,648	2,500	30.4	AXVL00000000	JGI-PGF
6	*Leptotrichia wadei*	Gram-negative rods, facultative, non-motile, non-spore-forming, isolated from saliva of a healthy person	2.35345	22,115	2,069	29.3	ARDS00000000	JGI-PGF
7	*Leptotrichia* sp. oral taxon 879 str. F0557	Isolates from a population of *Leptotrichia*, clearly distinct from currently recognized species. Tentatively designated at the species level. Unnamed isolates have not yet been characterized using traditional methods, and the species name has not yet been validly published.	2.41575	2,293	2,177	29.7	AWVL00000000	NCBI
8	*Leptotrichia* sp. oral taxon 215 str. W9775	Isolates from a population of *Leptotrichia*, clearly distinct from currently recognized species. Tentatively designated at the species level. Unnamed isolates have not yet been characterized using traditional methods, and the species name has not yet been validly published.	2.30849	2,158	2,052	31.4	AWVR01000000	NCBI
9	*Leptotrichia* sp. *Marseille*-P3007	*Leptotrichia massiliensis* was isolated from sputum in a healthy patient as part of a ‘culturomics’ study aiming at cultivating all bacteria in human stool	2.53864	2,388	2,307	29.7	NZ_FNVZ00000000	NCBI
10	*Leptotrichia* sp. oral taxon 212 str. W10393	Isolates from a population of *Leptotrichia*, clearly distinct from currently recognized species. Tentatively designated at the species level. Unnamed isolates have not yet been characterized using traditional methods, or the species name has not yet been validly published.	2.4449	2,289	2,159	31.4	CP012410	NCBI
11	*Leptotrichia* sp. oral taxon 847 str.F0260	Isolates from a population of *Leptotrichia*, clearly distinct from currently recognized species. Tentatively designated at the species level. Unnamed isolates have not yet been characterized using traditional methods, or the species name has not yet been validly published.	2.19494	2,070	1,939	29.8	CP014231	NCBI
12	*Leptotrichia* sp. oral taxon 225 str. F0581	Isolates from a population of *Leptotrichia*, clearly distinct from currently recognized species. Tentatively designated at the species level. Unnamed isolates have not yet been characterized using traditional methods, and the species name has not yet been validly published.	2.40008	2,248	2,155	29.6	AWVS00000000	NCBI

Table adopted and modified from Gupta et al. [100].

JGI-PGF, US DOE Joint Genome Institute; NCBI, www.ncbi.nlm.nih.gov/genome/genomes/14961?

**Table 2.** Update on reported *Leptotrichia* infections. Cases 1–54 were reported in a previous review by the authors [2]

Case	Sex (years)	*Leptotrichia* species identified	Clinical-associated disease/predisposing factors/recovery	Suggested source and port of entry (type of material)	Other microbes isolated/Identification type	Reference
55	US	*Leptotrichia* spp., *L. wadei*, *L. goodfellowii*, *L. trevisanii, L. hongkongensis*, *L. buccalis*	Wounds, respiratory, amniotic fluid, multiple myeloma, neutropenic fever, mucositis, HSCT, R	Blood, wounds, respiratory, amniotic fluid, (bacteremia)	Polymicrobial containing pathogens (viridans group streptococci, *E. faecium*, *B. urealyticus*, *Streptococcus, S. infantis*, or *F. nucleatum*), culture, DNA sequencing	[4]
56	F 74	*L. trevisanii*	Immunocompetent, pharyngeal pain, RSRTIW, normal renal function, AML, MLD, PA, oral lesion, fever, pneumonia, R, SD	Blood (bacteremia)	*Enterococcus faecium*, *S. epidermidis*, fungus, culture, 16S rRNA gene sequencing	[5]
57	F 66	*L. hongkongensis*	Metastatic breast carcinoma, cataract, lesions of lungs, pleura, lymph node, metastatic bilateral retinal detachment, fever, colonic polyp, R	Blood, mouth (bacteremia)	Culture, 16S rRNA gene sequencing, *groEL*, *gyrB*, *recA, rpoB* genes	[6]
58	M/F 52.3 ± 22.3	*Leptotrichia* spp.	Liver abscess, neutropenic sepsis, mucositis, HF, AML, intraabdominal, oropharyngeal and pelvic, cholangiocarcinoma, some D, some R	Blood (bacteremia)	*Propionibacterium* spp., *Bacteroides* spp., *M. morganii*, *D. pneumosintes*, *E. faecalis*, *B. fragilis*, *K. oxytoca*, *Prevotella* spp., *E. coli*, *Clostridium* spp., *C. perfringens*, *C. tertium*, *Fusobacterium* spp., anaerobic Gram-positive cocci, cultures, 16S rRNA gene sequencing	[7]
59	US	*L. buccalis*	Root canals, open cavities, provoked pain, sinus tract, palpation	Root canals	*E. faecalis*, *C. gracilis*, *E. saburreum*, *P. melaninogenica*, *T. socranskii*, *M. micros*, *P. gingivalis*, *P. endodontalis*, *P. nigrescens*, *S. anginosus*, *F. nuc*. ssp. *vincentii*, *F. nuc*. ssp. *nucleatum*, *V. parvula*, *N. mucosa*, checkerboard DNA–DNA hybridization	[8]
60	M 53	*L. trevisanii*	PBSCT, myeloblative chemotherapy, NHL, NF, relapsed follicular, mucositis, multiple myeloma, R	Blood (bacteremia)	*Sphingomonas paucimobilis*, cultures, RapID ANA II, Vitek, 16S rRNA gene sequencing	[9]
	M 56	*L. trevisanii*	PBSCT, multiple myeloma, NHL, mucositis, NF, myeloablative chemotherapy, relapsed follicular, R	Blood (bacteremia)	*Sphingomonas paucimicrobilis*, cultures, RapID ANA II, Vitek, 16S rRNA gene sequencing	
	F 63	*L. trevisanii*	PBSCT, AML, MPS, NF, NR, myeloablative chemotherapy, mucositis, R	Blood (bacteremia)	*Sphingomonas paucimicrobilis*, cultures, RapID ANA II, Vitek, 16S rRNA sequencing	
63	F 12	*L. trevisanii*	AML, mandible tumor, stomatitis, PBSCT, NF, chemotherapy, R	Blood (bacteremia)	*Sphingomonas paucimobilis* culture, RapID ANA II, Vitek, 16S rRNA gene sequencing	[10]
	M 66	*L. trevisanii*	Esophageal carcinoma, chemotherapy, NF, dysphagia, esophageal lesion, R	Blood (bacteremia)	*Tissierella praeacuta*, culture, RapID ANA II, Vitek, 16S rRNA gene sequencing	
65	M 78	*L. goodfellowii*	Immunocompetent, dyspnea, nausea, HF, DI, BC, hypertension, periumbilical pain, bilateral opacities, lung lesion, bronchopneumonia, fever, pulmonary edema, R	Blood (bacteremia)	Cultures, VMS, MALDI-TOF MS, 16S rRNA gene sequencing	[11]
66	M 44	*L. goodfellowii*	Immunocompetent, bioprosthetic pulmonic valve, headaches, aortic valve homograft, fever, infective endocarditis, chronic night sweats (diaphoretic), fatigue, inflammatory markers (ESR and CRP), elevated R	Blood (bacteremia)	Culture, GC, RapID ANAII test, 16S rRNA gene sequencing	[12]
67	M 55	*L. trevisanii*	Myelodysplastic syndrome, fever, trisomy, nausea, stomatitis, gum bleeding, mucositis, chemotherapy, neutropenic, pancytopenia, R	Blood (bacteremia)	Cultures, 16S rRNA gene sequence	[14]
68	F 80	*L. buccalis*	Subacute dyspnea, AML, mucositis, malaise, mild PBSB, thrombocytopenia, neutropenic fever, moderate normocytic anemia, blood transfused, R	Blood (bacteremia)	Gram-negative rod, cultures, 16S rRNA gene sequencing	[15]
69	M/F 2–97	*Leptotrichia* spp.	Coronary artery disease, candidal esophagitis, DI, DU, EG, GRD, GU, HH, RE, IMS, chronic kidney disease, UGIB, RT, sarcoidosis	Gastric fluid	*Lactobacillus* spp., Bacteroidetes, *Fusobacterium* spp., Proteobacteria, *R. dentocariosa*, Firmicutes, Actinobacteria, *A. odontolyticus*, *Prevotella, H. pylori*, *C. concisus*, *C. albicans*, *C. parapsilosis*, *C. tropicalis*, *P. pneumonia*, qPCR, HTS	[16]
70	F 69	*L. trevisanii*	Diffuse large B-cell lymphoma, mucositis, febrile diarrheal syndrome, catheter-related bloodstream infection, post-transplant aplasia, febrile, blood progenitor-cell transplantation, R	Stool, blood (bacteremia)	Culture, MALDI-TOF MS, 16S rRNA gene sequencing	[17]
71	M/F 71.1	*L. hongkongensis*, *Leptotrichia* spp., *Leptotrichia* sp. oral taxon	Pancreatic cancer	Saliva, mouth	*Porphyromonas*, *Bacteroides, Neisseria*, qPCR, HTS	[18]
	M/F 64.7	*Leptotrichia* spp.	Other disease (including cancer)	Saliva (mouth)	*Porphyromonas, Bacteroides*, qPCR, HTS	
	M/F 54.8	*Leptotrichia* spp.	Healthy	Saliva (mouth)	*Porphyromonas, Bacteroides*, qPCR, HTS	
74	M/F 62–66	*Leptotrichia* spp.	HNSCC, OPSCC-HPV negative	Tumor tissues, saliva	*Streptococcus*, *Peptostreptococcus, Staphylococcus*, *Neisseria, Haemophilus*, *Eikenella*, *Citrobacter*, *Parvimonas, Tannerella*, *Lactobacillus, Prevotella*, qPCR, HTS	[19]
	M/F 62–66	*Leptotrichia* spp.	HNSCC, OPSCC-HPV positive	Tumor tissues, saliva	*Streptococcus, Peptostreptococcus, Weeksellaceae*, *Tannerella, Parvimonas*, *Staphylococcus, Prevotella*, *Lactobacillus, Veillonella*, qPCR, HTS	
	M/F 62–66	*Leptotrichia* spp.	HNSCC, OSCC-HPV negative	Tumor tissues, saliva	*Streptococcus*, *Peptostreptococcus, Lactobacillus*, *Haemophilus, Neisseria*, *Parvimonas, Staphylococcus*, *Prevotella, Tannerella*, *Eikenella*, qPCR, HTS	
	M/F 62–66	*Leptotrichia* spp.	HPV negative, NM	Tumor tissues, saliva	*Streptococcus*, *Prevotella, Lactobacillus*, *Haemophils, Gemella*, *Neisseria, Aggregatibacter*, *Lautropia, Eikenella*, qPCR, HTS	
78	F 62	*L. trevisanii*	Hematological disease, symptomatic myeloma, oral pain, multiple myeloma, relapsed, fever, persistent catarrhal, dyspnea, deep medullary aplasia, mucositis, febrile neutropenia, cough, allogeneic bone marrow transplant, R	Blood (bacteremia)	Cultures, MALDI-TOF, 16S rRNA gene sequencing	[13]
79	M/F 56.8 ± 13.9 (23.5–80.9)	*Leptotrichia* spp.	Hematological disease, AML, myeloma, gut hemorrhage, BMT, acute lymphoid leukemia, chronic lymphoid leukemia, myelodysplastic syndrome, lymphoma, neutropenia, decubitus ulcer, sacrum decubitus bedsore, amygdalitis, dental abscess, mucositis, necrotic gingivitis, sigmoiditis, pertonitis	Blood (bacteremia)	*Bacteroides* spp., *B. fragilis* group, *Fusobacterium* ssp., *Clostridium* spp., *C. tertium*, *Staphylococcus* spp., *E. coli*, *P. intermedia*, *Enterococcus faecium*, *E. aerogenes*, *S. anginosus*, *S. sanguinis*, *S. mitis*, *S. constellatus*, *K. oxytoca*, *K. pneumoniae*, culture, BacT/Alert 240 system	[22]
	M/F 64.9 ± 15.7 (35.1–85.7)		Non-hematological disease, decubitus ulcer, sacrum decubitus bedsore, dental abscess, gut ischemia, abdominal gunshot wound, vertebral osteomyelitis, amygdalitis, diverticulitis, biliary tract infection appendicitis, peritonitis	Blood	*Bacteroides* spp., *B. fragilis* group, *Peptostreptococcus* spp., *Clostridium* spp., *C. perfringens*, *Bifidobacterium* spp., *S. constellatus*, *S. anginosus*, *S. sanguinis*, *E. faecium*, *Staphylococcus* spp., *E. coli*, culture, BacT/Alert 240 system	
81	US	*L. goodfellowii*, *L. buccalis*, *Leptotrichia* spp., uncultured *Leptotrichia* sp. oral clone	Guinea pigs	Oral swab samples	*Streptobacillus moniliformis*, uncultured bacterium, PCR amplicons, DNA sequencing	[21]
82	M/F 82–92 ± 85.6	*Leptotrichia* spp.	Root caries	Plaque	*Actinomyces*, *Selenomonas* sp. clone, *S. sputigena*, *Propionibacterium* spp., *P. alactolyticus*, *Actinomyces* sp. clone, *Prevotella* spp., *Veillonella, Veillonella* sp. clone, *V. parvula*/*V. dispar*, *F. nuc*. ssp. *polymorphum*, *Streptococcus* spp., *S. gordonii*, *S. intermedius*, *S. mutans*, *C. matruchotii*, *Atopobium, E. faecalis*, *L. casei*/*L. paracasei*/*L. rhamnosus*, *Olsenella* spp., cloning, 16S rRNA gene sequencing	[23]
	M/F 86–98 ± 91.8	*Leptotrichia* spp.	Healthy	Supragingival plaque	*P. melaninogenica*, *P. conceptionensis*, *Selenomonas* sp. clone, *S. sputigena*, *S. noxia*, *F. nuc*. ssp. *polymorphum*, *Veillonella* spp., *Actinomyces, K. oralis*, *C. matruchotii*, *C. gracilis*, *C. curvus*, *S. gordonii*, *S. mutans*, *S. mitis* bv. 2, *S. cristatus*, *S. anginosus*, cloning, 16S rRNA gene sequencing	
84	M 73	*L. wadei*, *Leptotrichia* spp.	Immunocompetent, pneumonia, hypoxemia, sore throat, fever, dyspnea, cough, leukocytosis, R Note: 1st case of pneumonia	BALF, mouth-gargled water	*Staphylococcus* spp., *Acidaminococcus* spp., *Veillonella* spp., *V. parvula*, *V. atypica*, *V. dispar*, *Lactobacillus* spp., *Enterococcus* spp., *E. faecalis*, *E. casseliflavus*, *P. nanceiensis*, culture, cloning, 16S rRNA gene sequencing	[24]
85	M/F 3–6	*Leptotrichia*	Caries-free male, male with caries, caries-free females, female with caries	Saliva, plaque	*Capnocytophaga*, *Peptostreptococcus, Corynebacterium*, *Rothia, Veillonella*, *Prevotella, Granulicatella*, *Streptococcus, Actinomyces*, *Thiomonas, Kingella*, *Campylobacter, Fusobacterium*, *Erysipelothrix, Atopobium*, *Oribacterium, Haemophilus*, *Neisseria*, DGGE, HTS	[25]
86	M/F 3–5	*Leptotrichia* spp.	Moderate caries	Plaque	*Capnocytophaga*, *Corynebacterium, Campylobacter*, *Haemophilus, mitis* group streptococci, *mutans* group streptococci, *Neisseria, Burkholderia*, *Actinomyces, Prevotella*, DGGE, cloning, 16S rRNA gene sequencing	[26]
	M/F 3–5	*Leptotrichia* spp.	Caries-susceptible	Plaque	*Capnocytophaga, Corynebacterium, Actinomyces*, *Burkholderia, mutans* group streptococci, *Neisseria, Haemophilus*, *Prevotella*, DGGE, cloning, 16S rRNA gene sequencing	
	M/F 3–5	*Leptotrichia* spp.	Caries-free	Plaque	*Mitis* group streptococci, *mutans* group streptococci, *Neisseria, Prevotella*, *Campylobacter, Burkholderia*, *Capnocytophaga, Corynebacterium*, *Actinomyces, Haemophilus*, DGGE, cloning, 16S rRNA gene sequencing	
89	M/F 25–39	*Leptotrichia* spp.	Unhealthy, gingivitis	Plaque, saliva	*S. sanguinis*, *Veillonella, Prevotella*, *Neisseria, Fusobacterium*, *Rothia*, TM7, *H. parainfluenzae*, *Granulicatella, L. mirabilis*, *Selenomonas, Actinomyces*, HTS, PCA	[27]
	F 21–23 ± 18	*Leptotrichia* spp.	Healthy	Plaque, saliva	*S. sanguinis, Veillonella, Prevotella, Neisseria, Granulicatella*, *Selenomonas, Rothia*, *L. mirabilis*, *Actinomyces, H. parainfluenzae*, *Fusobacterium*, HTS, PCA	
91	F 19–89	*Leptotrichia* spp.	Healthy, oral cancer, premalignant oral lesions	Saliva	*Streptococcus*, *Veillonella, Capnocytophaga*, *Haemophilus, Campylobacter*, *Atopobium, Mycoplasma*, *Lactococcus, Granulicatella*, *Filifactor, Prevotella*, *Parvimonas, Fusobacterium*, *Gemella, Kingella*, *Neisseria, Slakia*, 454 FLX-pyrosequencing, HOMIM DNA microarray	[28]
92	M/F 3–6 Mo	*Leptotrichia* spp.	Edentulous infants	Saliva	*Streptococcus*, *Haemophilus, Veillonella*, *Treponema, Gemella*, *Prevotella, Fusobacterium*, *Actinomyces, Granulicatella*, *Porphyromonas, Oribacterium*, *Campylobacter, Neisseria*, *Rothia*, HTS	[29]
93	F ≤ 20, 21–30, ≥31	*Leptotrichia* spp.	Sexually active: young and old partners, HIV, vaginal discharge, candidiasis, trichomoniasis	Vaginal fluid	*Lactobacillus*, *Bifidobacterium, Dialister*, *Prevotella, Peptoniphilus* non-*lacrimalis*, *G. vaginalis*, *Sneathia, Mobiluncus*, *M. hominis*, *Eggerthella, A. vaginae*, *Lactobacillus, T. vaginalis*, *M. elsdenii*, PCR	[30]
94	M 20–40	*Leptotrichia* spp.	Left skin feet	Skin emanation samples	*Staphylococcus* spp., *Corynebacterium* spp., *Propionibacteria* spp., *Delftia* spp., *Bacillus* spp., *Pseudomonas* spp., *Brevibacterium* spp., *Actinobacteria* Gp3 spp., *Variovorax* spp., *Micrococcus* spp., culture, 16S rRNA gene sequencing	[31]
95	F 17–21	*Leptotrichia* spp.	Sexually inactive: no sexual contact, vaginal discharge, or odor	Vaginal swab smear	*G. vaginalis*, *Megasphaera, Atopobium vaginae*, qPCR	[32]
	F 17–21	*Leptotrichia* spp.	Sexually active: no penile vaginal sex, vaginal discharge, or odor	Vaginal swab smear	*G. vaginalis*, *Sneathia, Megasphaera*, *A. vaginae*, qPCR	
	F 17–21	*Leptotrichia* spp.	Sexually active: penile vaginal sex, vaginal discharge, or odor	Vaginal swab smear	*G. vaginalis*, *Sneathia, Megasphaera*, *A. vaginae*, qPCR	
98	M 81	*Leptotrichia* spp.	Immunocompetent, DI, cough, fever, fatigued, chills, RD, HSCT, CAP, dyspnea, lung cancer or vasculitis, rigors, cavity lesion, pneumonia, mild anemia, pulmonary diseases, bilateral lungs crackles, respiratory distress, R Note: 3rd case of pneumonia	Blood, bronchial wash fluid (bacteremia)	*S. aureus*, *Streptococcus* group B, viridans *Streptococcus*, culture	[33]
99	M/F 18–55 (35.6 ± 11.8)	*L. hofstadii*, *L. buccalis*, *L. wadei*, *L. shahii*, *Leptotrichia* spp.	Patients	Saliva, plaque, mucosal surfaces	*Streptococcus*, *S. mutans*, *Gemella, Corynebacterium*, *Cardiobacterium, G. elegans*, *Selenomonas, Porphyromonas*, *Campylobacter, Neisseria*, *Rothia, Prevotella*, *A. porcinus*, *Actinomyces, Veillonella*, *C. dublinensis*, *Lautropia*, DGGE, Cloning, 16S rRNA gene sequencing	[34]
	M/F 21–54 (35.9 ± 11.7)	*Leptotrichia* spp.	Healthy without prosthesis	Saliva, plaque, mucosal surfaces	*Streptococcus*, *Corynebacterium, Selenomona*, *Veillonella, Actinomyces*, *Gemella, Neisseria*, *Rothia*, DGGE, cloning16S rRNA gene sequencing	
101	M 39–42.5	*Leptotrichia* spp.	Chronic periodontitis, inflammation, bone loss, bleeding, peri-implantitis, suppuration	Submucosal: sulci or peri-implant crevice, supragingival plaque	*Propionibacter*, *Prevotella, Corynebacterium*, *Campylobacter, Lactococcus*, *Gemella, Rothia*, *Actinomyces, Burkholderia*, non-*mutans Streptococcus*, *S. mutans*, *Mycoplasma, Peptococcus*, *Eubacterium, Neisseria*, *Solobacterium, Porphyromonas*, *Pseudomonas, Escherichia*, *Johnsonella, Achromobacter*, *Butyrivibrio, Peptoniphilus*, *Catonella, Treponema*, *Kingella, Lactobacillus*, *Dialister, Chloroflexi*, *Megasphaera, Selenomonas*, HTS, PCA	[35]
	M 35.5–41	*Leptotrichia* spp.	Healthy, periodontal peri-implant	Supragingival plaque	*Propionibacter*, *Porphyromonas, Corynebacterium*, *Neisseria, Prevotella*, *Fusobacterium, Propionibacterium*, Synergistes, *Dialister*, *Streptococcus, S. mutans*, *Granulicatella, Campylobacter*, *Burkholderia, Selenomonas*, *Rothia, B. fibrisolvens*, *Peptococcus, Lactobacillus*, *Veillonella, Arthrobacter*, non-*mutans Eubacterium*, *Actinomyces, Lactococcus*, *Mycoplasma, Treponema*, *Catonella*, HTS, PCA	
103	F 32.01 ± 5.12	*Leptotrichia* spp.	Obese women, gestational DI	Breast milk	*Streptococcus*, *Streptococcus* group B, *Staphylococcus*, *Veillonella*, TM7, *Prevotella, Weisella*, *Leuconostoc, Lactococcus*, qPCR, HTS	[36]
	F 32.01 ± 5.12	*Leptotrichia* spp.	Healthy, normal-weight women, gestational DI	Breast milk	*Streptococcus*, *Streptococcus* group B, *Staphylococcus*, *Prevotella*, TM7, *Weisella, Leuconostoc*, *Lactococcus, Veillonella*, qPCR, HTS	
105	F 42.2 ± 40	*Leptotrichia* spp., *L. wadei*	New-onset rheumatoid arthritis	Mouth	*Porphyromonas* clones, *P. gingivalis*, *Prevotella* spp., *Treponema* clones, *Streptococcus, Tannerella* clones, *Anaeroglobus geminatus*, *Neisseria, Selenomonas*, *Corynebacterium*, HTS, PCA, ELISA	[20]
	F 47.7 ± 48		Chronic established rheumatoid arthritis	Mouth	*P. gingivalis*, *Corynebacterium, Streptococcus*, *Selenomonas, Prevotella*, *A. geminatus*, *Treponema* clones, *Tannerella* clones, HTS, PCA, ELISA	
	F 42.2 ± 39		Healthy	Mouth	*P. gingivalis*, *Capnocytophaga, A. geminatus*, *Selenomonas, Prevotella*, HTS, PCA, ELISA	
108	M 37.08 ± 14.1		Severe dentin caries, biofilm	Carious lesions	*P. acidifaciens*, *S. mutans*, *L. homohiochii*, *L. rhamnosus*, *L. vaginalis*, *L. zeae*, *L. casei*, *L. lactis*, *L. pontis*, *L. panis*, *L. oris*, *L. frumenti*, qPCR	[37]
	M 32.28 ± 10.0	*Leptotrichia* spp., *L. wadei*, *L. trevisanii*, *Leptotrichia* sp. oral taxon	Caries-free, biofilm	Plaque	*P. acidifaciens*, *E. brachy*, *S. parasanguinis*, *S. sanguinis, S. constellatus*, *S. gordonii*, *S. mitis*, *S. anginosus*, *S. pneumoniae*, *S. australis*, *S. intermedius*, *S. oralis*, *G. morbillorum*, *Capnocytophaga* sp. oral taxon, *Capnocytophaga* spp., *C. sputigena*, *Treponema* spp., *Treponema* sp. oral taxon, *T. denticola*, *Fusobacterium* spp., *F. nucleatum*, *F. periodonticum*, qPCR	
110	M/F 22–24	*Leptotrichia* spp.	Healthy	Saliva	*Streptococcus*, *Lachnospiraceae, Peptostreptococcus*, *Flavobacteriaceae, Aggregatibacter*, *Porphyromonas, Corynebacterium*, *Granulicatella, Rothia*, *Eubacterium, Veillonella*, *Fusobacterium, Oribacterium*, *Neisseria, Gemella*, *Pasteurella, Prevotella*, *Actinomyces, Haemophilus*, *Moraxella*, SR1, HTS	[38]
	M/F 3–6	*Leptotrichia* spp.	Healthy	Saliva	*Streptococcus, Lachnospiraceae, Granulicatella*, *Fusobacterium, Neisseria*, *Aggregatibacter, Actinomyces*, *Haemophilus, Porphyromonas*, *Pasteurella, Rothia*, *Veillonella, Oribacterium*, *Gemella, Prevotella*, HTS	
112	1–60 days	*Leptotrichia* spp.	Fermenting Lees liquor	Liquor	*Corynebacterium*, *Staphylococcus, Microbacterium*, *Lactobacillus, Bacillus*, *Clostridium, Streptococcus*, *Burkholderia, Actobacter*, *Serratia, Rhodoccous*, *Pelobacter, Arthrobacter*, *Curtobacterium, Methanoculleus*, *Saccharomyces, Aspergillus*, *Eurotium, Zygosaccharomyces*, *Saccharomycopsis, Fomitopsis*, *Pichia, Talaromyces*, *Trichosporon*, 16S rRNA- and 18S rRNA gene sequencing	[39]
113	UK	*Leptotrichia* spp., *L. hofstadii*	Tumor tissue	Tumor tissues	*Campylobacter*, *Fusobacterium* spp., *F. nucleatum*, *C. showae*, *Ralstonia, Selenomonas*, *S. sputigena*, *Bacteroides*, HTS, PCA	[40]
	UK		Unaffected tissue	Surgical samples	*Ruminococcus*, *Pseudoflavonifractor, Ruminococcaceae*, *Parabacteroides, Bacteroides*, *Holdemania, Ralstonia*, HTS, PCA	
115	M/F 20–66	*Leptotrichia* spp.	TB	Sputum	Unclassified *Enterobacteriaceae*, *Veillonella, P. melaninogenica*, *Neisseria, Fusobacterium*, *Streptococcus*, *S. anginosus*, *S. mitis* clone, *Mogibacterium, Moryella*, *P. micra*, *Oribacterium, Prevotella*, *Pseudomonas, Lactococcus*, *L. crispatus*, *Actinomyces*, HTS, PCA	[41]
	M/F 22–82		TB-free	Sputum	*Streptococcus, S. parasanguinis* clone, unclassified *Lactobacillales*, *A. aphrophilus*, *Prevotella, Neisseria*, HTS, PCA	
117	M/F 19–47	*Leptotrichia* spp., *L. wadei*	Malodor individuals	Tongue plaque	*Prevotella*, *P. tannerae*, *Streptococcus, Fusobacterium*, *Veillonella, Gemella*, *Granulicatella, Neisseria*, *Rothia, Porphyromonas*, *Haemophilus, Actinomyces*, H_2_S, HTS, PCA	[42]
118	M/F 13–77 ± 44	*Leptotrichia* spp.	NTB, R	Sputum	*Mycobacterium*, *Streptococcus, Granulicatella*, *Haemophilus, Pseudomonas*, *Neisseria, Bergeyella*, *Acinetobacter, Haloplasma*, *Veillonella, Coprococcus*, *Alcaligenes, Treponema*, *Lautropia, Bulleidia*, *Prevotella, Catonella*, *Sharpea*, HTS	[43]
	M/F 22–79 ± 52		RTB, R	Sputum	*Mycobacterium*, *Granulicatella, Corynebacterium*, *Sharpea, Achromobacter*, *Stenotrophomonas, Pseudomonas*, *Streptococcus, Lactobacillus*, *Neisseria, Treponema*, *Bergeyella, Prevotella*, *Veillonella, Haloplasma*, *Coprococcus, Catonella*, *Alcaligenes, Rothia*, *Lautropia*, HTS	
	M/F 20–78 ± 49	*Leptotrichia* spp.	TFTB, failed	Sputum	*Mycobacterium*, *Streptococcus, Granulicatella*, *Campylobacter, Prevotella*, *Pseudomonas, Veillonella*, *Bergeyella, Haloplasma*, *Coprococcus, Sharpea*, *Atopobium, Blastobacter*, *Alcaligenes, Catonella*, *Treponema, Neisseria*, *Lautropia*, HTS	
	M/F 24–55 ± 31	*Leptotrichia* spp.	Healthy	Throat	*Granulicatella*, *Streptococcus, Campylobacter*, *Anaeroglobus, Pseudomonas*, *Treponema, Coprococcus*, *Haemophilus, Selenomonas*, *Bulleidia, Neisseria*, *Haloplasma, Atopobium*, *Prevotella, Clostridium*, *Catonella*, HTS	
122	M/F 50 (±47.5–52.5)	*Leptotrichia* sp. clones	Dental caries, dental caries + periodontitis	Saliva, caries lesions, mouth	*V. atypica*, *V. parvula*, *M. micronuciformis*, *F. periodontium*, *S. moorei*, *A. xylosoxidans*, *S. parasanguinis* sp. clones, *S. salivarius*, *S. salivarius* sp. clone, PCR, HOMIM	[44]
	M/F 55 (±53.8–56.3)	*Leptotrichia* sp. clones	Healthy and diseases (caries + periodontitis)	Saliva, mouth	*A. xylosoxidan*, *M. micronuciformis*, *F. periodontium*, *V. atypica*, PCR, HOMIM	
124	M/F < 30 Mo ±19.1	*Leptotrichia* spp., *L. hongkongensis* clones	Caries	Supragingival plaque, mouth	*Porphyromonas*, *Corynebacterium, Capnocytophaga*, *Streptococcus* spp., *S. mutans* clones, *S. sobrinus* clones, *Veillonella, Neisseria*, *Rothia*, TM7 genus *incertae sedis*, *Actinomyces, Prevotella* spp., *P. histicola* clones, *Eikenella, Kingella*, *Fusobacterium, Gemella*, *Campylobacter, Granulicatella* spp., *G. adiacens* clones, *Abiotrophia, Selenomonas*, *Acinetobacter, Lactobacillus*, *Anaeroglobus, Ottowia*, *Schlegelella*, HTS, PCA	[45]
	M/F < 30 Mo ±19.0	*Leptotrichia* spp.	CF	Supragingival plaque, mouth	*Streptococcus* spp., *Capnocytophaga, Corynebacterium*, TM7 genus *incertae sedis*, *Porphyromonas, Granulicatella*, *Fusobacterium, Treponema*, *Gemella, Selenomonas*, *Veillonella, Dechloromonas*, *Actinomyces, Campylobacter*, *Abiotrophia*, *Ottowia*, *Eikenella, Johnsonella*, *Neisseria, Prevotella*, *Kingella, Rothia*, HTS, PCA	
126	M/F/T 39 ± 10	*Leptotrichia* spp.	HIV seropositive	Saliva	*Lactobacillus* spp., *Aggregatibacter, Lachnospiraceae*, *Rothia, Eubacterium*, *Tannerella, Haemophilus*, *Neisseria, Gemella*, *Granulicatella, Shuttleworthia*, *Streptococcus, S. mutans*, *Fusobacterium, Solobacterium*, *Campylobacter, Veillonella*, *Dialister*, Synergistetes, *Filifactor, Parvimonas*, *Achromobacter*, Megasphaera, *Selenomonas, Prevotella*, *Candida*, culture, DGGE, HOMIM, PCA	[46]
	M/F 43 ± 13	*Leptotrichia* spp.	HIV seronegative	Saliva	*Capnocytophaga*, *Lachnospiraceae, Peptostreptococcaceae*, *Granulicatella, Veillonella*, Synergistetes, *Lactobacillus* spp., *Porphyromonas, Lactobacillus*, *Campylobacter, Streptococcus*, *S. mutans*, *Parvimonas, Kingella*, *Atopobium, Selenomonas*, *Aggregatibacter, Fusobacterium*, *Haemophilus, Megasphaera*, *Prevotella, Solobacterium*, *Gemella, Achromobacter*, *Eubacterium, Rothia*, *Slackia, Filifactor*, *Dialister, Neisseria*, *Candida*, culture, DGGE, HOMIM, PCA	
128	M/F ≥ 18–21.5 ± 1.9	*Leptotrichia* spp., *L. hongkongensis*	Healthy, supragingival plaque	Mouth	*Corynebacterium*, *Capnocytophaga, Streptococcus*, *Cardiobacterium*, *Haemophilus*, *Derxia, Veillonella*, *Prevotella*, HTS	[47]
129	UK	*Leptotrichia* spp., *L. wadei*	Healthy, biofilms	Oral epithelial cells	*Prevotella*, *Streptococcus* spp., qPCR	[48]
130	M/F 3–3 MY		Caries individuals	Plaque, biofilm, saliva, mouth	*Aggregatibacter* sp. HOT 513, *Streptococcus* genus, *S. oralis*, *S. mutans*, *S. sobrinus*, *S. mitis*/*S. mitis* bv2/*S. infantis*, *Streptococcus* sp. HOT 431, *Lactobacillus*, *Atopobium* genus, *A. parvulum*, *Actinobaculum* sp. HOT 513, culture, HTS, microarray	[49]
	M/F 3–3 MY	*L. hofstadii /Leptotrichia* sp. HOT 203 or 234	Healthy, CF	Plaque, biofilm, saliva, mouth	*C. concisus*, *G. adiacens*, *Actinomyces* sp. HOT 177, *Actinomyces* genus, *Kingella* genus, *K. dentificans*, *K. oralis*, *Streptococcus anginosus*/*S. gordonii*, *S. sanguinis*, *Bergeyella* sp. HOT 322, culture, HTS, microarray	
132	M 73–83 ± 77	*Leptotrichia* spp.	Lung, AECOPD, cough, dyspnea, fatigue, sputum production	Sputum	*Capnocytophaga*, *Stenotrophomonas, Pasteurellaceae*, *Pediococcus, Rothia*, *Acinetobacter, Porphyromonas*, *Streptococcus, Actinomyces*, *Enterobacter, Veillonella*, *Prevotella, Neisseria*, fungi (*Sterigmatomyces*, *Teratosphaeria, Candida*, *Aspergillus Phialosimplex*, *Aureobasidium*), 16S rRNA gene sequencing, barcoded ITS genes, HTS, CRP	[50]
133	UK/A	*Leptotrichia* spp.	Healthy, ciprofloxacin group	Saliva, fecal	*Veillonella*, *Bacteroides, K. pneumoniae*, *E. coli*, culture, microarray, MALDI-TOF, PFGE	[51]
	UK/A	*Leptotrichia* spp.	Healthy, clindamycin group	Saliva, fecal	*Veillonella*, *K. pneumoniae*, culture, MALDI-TOF, microarray, PFGE	
	UK/A	*Leptotrichia* spp.	Healthy, placebo control group	Saliva, fecal	*Veillonella*, *Bacteroides, K. pneumoniae*, culture, MALDI-TOF, microarray, PFGE	
136	M/F 48	*L. buccalis*	Rheumatoid arthritis patients, healthy subjects without periodontitis	Subgingival plaque samples	*P. gingivalis*, *T. forsythia*, *T. denticola*, checkerboard DNA–DNA hybridization	[52]
	M/F 48	*L. buccalis*	Rheumatoid arthritis patients, periodontitis, gingivitis	Subgingival plaque samples	*P. gingivalis*, *T. forsythia*, *T. denticola*, *G. morbillorum*, *S. gordonii*, *P. acnes*, checkerboard DNA–DNA hybridization	
138	M/F 3–6	*Leptotrichia* spp.	Healthy, intact enamel surface: discordant caries twins	Supragingival plaque, mouth	*Fusobacterium*, *Corynebacterium*, *Porphyromonas*, *Veillonella*, TM7 genus *incertae sedis*, *Streptococcus, Moraxella*, *Capnocytophaga, F. canifelinum*, *Selenomonas, Propionibacterium*, *Actinomyces, Neisseria*, *K. denitrificans*, *Alysiella, Prevotella*, *Lactobacillus, Scardovia*, HTS, PCA	[53]
	M/F 3–6	*Leptotrichia* spp.	Caries, intact enamel surface: discordant caries twins	Supragingival plaque, mouth	*Capnocytophaga*, *Propionibacterium, Streptococcus*, *Porphyromonas*, TM7 genus *incertae sedis*, *Prevotella, Lactobacillus*, *Moraxella, Selenomonas*, *Alysiella, Scardovia*, *Neisseria*, HTS, PCA	
	M/F 3–6	*Leptotrichia* spp.	Caries, decayed tooth surface: discordant caries twins	Supragingival plaque, mouth	*Propionibacterium*, *Corynebacterium, Capnocytophaga*, *Streptococcus, C. matruchotii*, *Veillonella, V. dispar*, *Prevotella, Porphyromonas*, TM7 genus *incertae sedis*, *Lactobacillus, Alysiella*, *Actinomyces, Selenomonas*, *S. noxia*, *Moraxella, Scardovia*, *Neisseria*, HTS, PCA	
141	M/F 4–21 DO	*Leptotrichia* spp.	PEDV	Piglets feces	Actinobacteria, *Verrucomicrobia*, Proteobacteria, Fusobacteria, Firmicutes, Bacteroidetes, MST, qPCR, 16S rRNA gene sequencing	[54]
	F 18–60+	*Leptotrichia* spp.	hrHPV, HIV+, HIV–	Vaginal swab suspensions	Proteobacteria, *Peptostreptococcus*, Bacteriodetes, *Peptoniphilus* spp., *L. iners*, *L. crispatus*, *Fusobactium* spp., *Atopobium, Bacillus*, *G. vaginalis*, *Megasphaera* spp., *Sneathia* spp., *Prevotella* spp., *Clostridia, Dialister* spp., HTS, PCA	[55]
	F 18–60+	*Leptotrichia* spp.	Negative hrHPV, HIV+, HIV–	Vaginal swab suspensions	*Prevotella* spp., Proteobacteria, *L. iners*, *L. crispatus*, *G. vaginalis*, HTS, PCA	
144	M 17	*L. buccalis*, *L. goodfellowii*, *L. shahii*, *L. hofstadii*, *L. wadei*, *L. hongkongensis*, *Leptotrichia* sp. clones	Active caries, caries lesions	Plaque, mouth, Swedish	*Peptostreptococcaceae*, *Porphyromonas, S. mutans*, *S. australis*, *S. mitis*, *D. pneumosintes*, *Capnocytophaga* spp., *Capnocytophaga* sp. clone, TM7 clone, *F. nuc*. ssp. *animalis*, *Lachnoanaerobaculum, Alloprevotella*, *Actinobaculum, Neisseria*, *Kingella, Eubacterium* spp., *G. haemolysans*, *Selenomonas, P. oris*, *P. maculosa*, *P. nigrescens*, *Treponema, A. gerencseriae*, *Actinomyces* sp. clone, *Parvimonas*, Bacteroidales, *C. matruchotii*, *Bergeyella, Veillonella*, *Mitsuckella*, PCR, qPCR, HTS	[56]
	M 17	*L. buccalis*, *L. goodfellowii*, *L. shahii*, *L. hofstadii, L. wadei*, *L. hongkongensis*, *Leptotrichia* sp. clones	Healthy, CF	Plaque, mouth, Swedish	*Streptococcus* spp., *Capnocytophaga* sp. clone, *Capnocytophaga* spp., *F. nuc*. ssp. *animalis*, *Campylobacter, S. mutans*, *Actinomyces* sp. clone, *P. maculosa*, *P. nigrescens*, *Actinomyces, Selenomonas*, Clostridiales clones, *Dialister, Mycoplasma*, PCR, qPCR, HTS	
	M 14–15	*L. buccalis, L. hongkongensis, L. shahii*, *L. goodfellowii*, *L. wadei, L. hofstadii*, *Leptotrichia* sp. clones	High caries	Plaque, mouth, Romania	*Peptostreptococcus*, *Lachnospiraceae* clone, *Capnocytophaga, Catonella*, *D. pneumosintes*, *S. sobrinus*, *S. australis*, *S. sanguinis*, *S. sinensis*, *S. cristatus*, *S. mutans*, *S. mitis*, *Streptococcus* sp. clones, *Fusobacterium*, *G. haemolysans*, *Filifactor, Actinomyces*, *Shuttleworthia*, *Campylobacter*, *Ganulicatella*, TM7 clone, *Abiotrophia, P. catonella*, Bacteroidetes clone, *Parvimonas*, *Neisseria*, *Selenomonas*, *Veillonella*, *Lactobacillus*, *Prevotella* spp., *Prevotella* sp. clone, *Alloprevotella* clone, PCR, qPCR, HTS	
147	M/F 55–74 (60.77–63.71)	*Leptotrichia* spp.	Prostate, lung, colorectal, and ovarian (PLCO-a) head and neck patients	Oral wash samples	*Corynebacterium*, *Bifidobacterium, Peptostreptococcus*, *Porphyromonas, V. parvula*, *Capnocytophaga, Selenomonas*, *Aggregatibacter, Lactobacillus*, *Kingella, Neisseria*, *Streptococcus, Eikenella*, *Haemophilus, Abiotrophia*, *Atopobium, Lautropia*, *Prevotella*, HTS	[57]
	M/F 55–74 (61.02–64.25)	*Leptotrichia* spp.	PLCO-b pancreas patients	Oral wash samples	*Corynebacterium*, *Bifidobacterium, Peptostreptococcus*, *Porphyromonas, V. parvula, Capnocytophaga, Selenomonas*, *Streptococcus, Aggregatibacter*, *Haemophilus, Lactobacillus*, *Prevotella, Abiotrophia*, *Eikenella, Lautropia*, *Neisseria, Atopobium*, *Kingella*, HTS	
	M/F 55–74 (68.82–70.53)	*Leptotrichia* spp.	Cancer Prevention Study II (CPS-II-a) head and neck patients	Oral wash samples	*Corynebacterium*, *Bifidobacterium, Peptostreptococcus*, *Porphyromonas, V. parvula*, *Streptococcus, Capnocytophaga*, *Aggregatibacter, Haemophilus*, *Atopobium, Abiotrophia*, *Selenomonas*, *Eikenella*, *Lactobacillus, Lautropia*, *Neisseria, Prevotella*, *Kingella*, HTS	
	M/F 55–74 (70.77–74.80)	*Leptotrichia* spp.	CPS-II-b pancreas patients	Oral wash samples	*Corynebacterium, Bifidobacterium, Peptostreptococcus, Porphyromonas, V. parvula, Streptococcus, Capnocytophaga, Aggregatibacter, Haemophilus, Atopobium, Selenomonas, Eikenella, Lautropia*, *Lactobacillus, Abiotrophia*, *Prevotella, Neisseria*, *Kingella*, HTS	
151	US	*Leptotrichia* spp.	Herbivorous, carnivorous, omnivorous, and fish filter-feeding	Fish gut	*Cetobacterium*, *Clostridium, Bacteroides*, *Shewanella, Xiphinematobacter*, *Citrobacter, Halomonas*, 16S rRNA gene sequencing, HTS, PCA	[58]
152	M/F 20–50	*Leptotrichia* spp.	Brush-alone, gingivitis, R	Plaque, saliva	*Actinomyces*, *Actinobaculum, Lachnospiraceae*, *Bergeyella, Granulicatella*, *Lautropia, Selenomonas*, *Prevotella, Tannerella*, uncultured *Peptococcus*, unclassified *Veillonellaceae*, TM7, *Rothia*, HTS, PCA, MA	[59]
	M/F 18–50	*Leptotrichia* spp.	Brush-plus-rinse, gingivitis, R	Plaque, saliva	*Actinomyces*, *Actinobaculum, Lachnospiraceae*, *Bergeyella, Granulicatella*, *Selenomonas, Tannerella*, *Lautropia, Peptococcus*, *Prevotella*, TM7, *Rothia*, unclassified *Veillonellaceae*, HTS, PCA, MA	
154	M/F 18–45 ± 27.3	*Leptotrichia*	Healthy, normal oropharyngeal and intestine, R	Blood, saliva, mouth,	*Bifidobacteria*, *Enterobacteria*, enterococci, lactobacilli, *Streptococcus* spp., *S. salivarius*, Fusobacteria, *Veillonella*, *Clostridia*, Staphylococci, Micrococci, *Neisseria*, *Prevotella, Candida*, culture, MALDI-TOF MS, GC, qPCR	[60]
	M/F 18–45 ± 27.3		Healthy, normal oropharyngeal and intestine, R	Fecal, blood	Enterococci, Enterobacteria, Bifidobacteria, *Bacteroides*, *Clostridia*, *E. coli*, lactobacilli, *Candida*, culture, MALDI-TOF MS, GC, qPCR	
156	M/F 41–60	*Leptotrichia* spp.	Cholelithiasis (gallstone disease), fish-borne liver fluke infection (*Opisthorchis felineus*), pancreatitis, hepatitis C virus	Aspirated bile	*T. socranskii*, *T. amylovorum*, *Aggregatibacter, Klebsiella*, *Flavobacterium, P. distasonis*, *P. aminovorans*, *L. brevis*, *V. dispar*, TG5, *C. durum*, *B. flexus*, *B. uniformis*, *R. aeria*, *H. influenza*, *H. parainfluenzae*, *S. equorum*, *Zoogloea, A. johnsonii*, *A. lwoffii*, *Cellulosimicrobium, Sediminibacterium*, *Dorea, Saccharopolyspora*, *Parabacteroides, S. changbaiensis*, *Phycicoccus, P. mexicana*, *Granulicatella, Halogeometricum* clone, *Selenomonas, M. mobilis*, *M. adhaesivum*, *Friedmanniella, Luteolibacter*, *Mycoplana, S. yabuuchiae*, *S. xenophagum*, *Microlunatus, Pimelobacter*, *Brochothrix, Ochrobacterum*, *Ruminococcus, Psychrobacter*, *S. anginosus*, *Lutibacterium, Oscillospira*, *Anaerostripes, Kaistobacter*, PCR, qPCR, HTS, PCA	[61]
157	M/F 20–50	*Leptotrichia* spp.	Low caries load	Supragingival plaque	*Porphyromonas*, *Capnocytophaga, Corynebacterium*, *Propionibacterium, Campylobacter*, *Streptococcus, Ottowia*, *Fusobacterium, Actinobaculum*, *Actinomyces, Selenomonas*, *Prevotella, Neisseria*, *Lautropia, Veillonella*, TM7, *Rothia*, HTS, PCA	[62]
	M/F 20–50	*Leptotrichia* spp.	Moderate caries load	Supragingival plaque	*Corynebacterium*, *Capnocytophaga, Propionibacterium*, *Ottowia, Neisseria*, *Campylobacter, Porphyromonas*, *Actinobaculum, Fusobacterium*, *Prevotella, Streptococcus*, *Selenomonas, Actinomyces*, *Veillonella, Lautropia*, *Rothia*, TM7, HTS, PCA	
	M/F 20–50	*Leptotrichia* spp.	High caries load	Supragingival plaque	*Capnocytophaga*, *Corynebacterium, Propionibacterium*, *Prevotella, Rothia*, *Neisseria, Fusobacterium*, *Porphyromonas, Campylobacter*, *Streptococcus, Actinomyces*, *Actinobaculum, Selenomonas*, *Lautropia*, TM7, *Veillonella*, HTS, PCA	
	M/F 20–50	*Leptotrichia* spp.	Healthy, CF	Supragingival plaque	*Cardiobacterium*, *Propionibacterium, Capnocytophaga*, *Fusobacterium, Corynebacterium*, *Aggregatibacter, Selenomonas*, *Porphyromonas, Ottowia*, *Actinomyces, Actinobaculum*, *Prevotella, Veillonella*, *Rothia, Campylobacter*, *Neisseria, Streptococcus*, TM7, *Lautropia*, HTS, PCA	
161	US	*Leptotrichia* spp.	Healthy, CF, no pigment	Supragingival plaque, saliva	*Neisseria*, unclassified *Neisseriaceae*, *Capnocytophaga, Parascardovia*, *Prevotella, Streptococcus*, unclassified *Streptococcaceae*, *Paenibacillus, Rothia*, *Haemophilus*, HTS, PCA	[63]
	US	*Leptotrichia* spp.	BPES patients	Supragingival plaque, saliva	*Neisseria*, unclassified *Neisseriaceae*, *Capnocytophaga, Mogibacterium*, *Granulicatella, Parascardovia*, *Prevotella, Fusobacterium*, *Streptococcus*, unclassified *Streptococcaceae*, Gemellales, *Prevotella*, *Paenibacillus, Rothia*, *Veillonella*, unclassified *Haemophilus*, HTS, PCA	
	US	*Leptotrichia* spp.	Active caries (obvious decay)	Supragingival plaque, saliva	*Neisseria*, unclassified *Neisseriaceae, Capnocytophaga, Granulicatella*, *Mogibacterium, Streptococcus*, unclassified *Streptococcaceae*, *Rothia, Gemella*, *Prevotella, Fusobacterium*, *Selenomonas, Veillonella*, unclassified Gemellales, *Streptobacillus, Paenibacillus*, *Parascardovia, Haemophilus*, HTS, PCA	
	US	*Leptotrichia* spp.	Active caries + pigment (obvious decay)	Supragingival plaque, saliva	*Streptococcus*, unclassified *Streptococcaceae*, *Peptostreptococcus, Neisseria*, unclassified *Neisseriaceae*, *Clostridium*, unclassified *Clostridiaceae*, *Rothia, Gemella*, unclassified Gemellales, *Granulicatella, Capnocytophaga*, *Selenomonas, Paenibacillus*, *Prevotella, Mogibacterium*, *Parascardovia, Fusobacterium*, *Streptobacillus, Haemophilus*, HTS, PCA	
165	M/F 18–60	*Leptotrichia* spp.	Behçet’s disease patients	Saliva	*H. parainfluenzae*, *Alloprevotella*, MiSeq sequencing	[64]
	M/F 22–54	*Leptotrichia* spp.	Healthy	Saliva	*Haemophilus*, *P. enoeca*, *Alloprevotella, Lachnospiraceae*, *L. orale*, TM7 sp., *Veillonella,* Bacteroidetes spp., Clostridiales, *C. concisus*, *Rothia, S. moorei*, *Selenomonas, P. pallens*, *A. graevenitzii*, *A. parvulum*, *Neisseria*, MiSeq sequencing	
167	M/F 4–5	*Leptotrichia* spp.	Halitosis	Supragingival plaque	*Actinomyces*, *Porphyromonas, Prevotella*, *Lautropia, C. ochracea*, *S. noxia*, HTS	[65]
	M/F 4–5	*Leptotrichia* spp.	Healthy	Supragingival plaque	*Prevotella*, *Actinomyces, Porphyromonas*, HTS	
169	M/F 50–74	*Leptotrichia* spp.	Cancer Prevention Study (CPS) II	Oral wash samples	*P. gingivalis*, *A. actinomycetemcomitans*, *T. forsythia*, *Alloprevotella, P. intermedia*, 16S rRNA gene sequencing, HOMD, PCA	[66]
	M/F 55–74	*Leptotrichia* spp.	Prostate, lung, colorectal, and ovarian cancer (PLCO)	Oral wash samples	*P. gingivalis*, *A. actinomycetemcomitans*, *T. forsythia*, *Alloprevotella, P. intermedia*, 16S rRNA gene sequencing, HOMD, PCA	
	M/F 63.8–73.1	*Leptotrichia* spp.	Nested case control with no prior history of cancer	Oral wash samples	*P. gingivalis, A. actinomycetemcomitans, T. forsythia, Alloprevotella, P. intermedia*, 16S rRNA gene sequencing, HOMD, PCA	
172	US	*Leptotrichia wade*, *Leptotrichia* spp.	Patient with caries cavity, supragingival plaque	Plaque samples, saliva mucosal swabs	Hemolytic bacterium, streptococci, *S. mutans*, *S. tigurinus*, *F. nucleatum*, *Lactobacillus, C. albicans*, Gram-stain, culture, VITEK system, qPCR, DGGE, 16S rRNA gene sequencing	[67]
	US	*Leptotrichia* spp.	Healthy	Plaque samples, saliva mucosal swabs	Gram-stain, culture, VITEK system, qPCR, DGGE, 16S rRNA gene sequencing	
174	F 18.3–36.3	*Leptotrichia*	Healthy	Maternal saliva, premasticated foods	*Sphingomonas*, unclassified *Pasteurellaceae*, *Porphyromonas, Eubacterium*, *Fusobacterium*, *Gemella*, *Veillonella, Johnsonella*, *Streptococcus, Neisseria*, *Actinomyces, Rothia*, *Prevotella*, MiSeq sequencing	[68]
	M/F 0.8–2.0	*Leptotrichia*	Healthy, breastfeeding	Infant saliva, premasticated foods	*Streptococcus*, *Fusobacterium, Porphyromonas*, *Sphingomonas, Gemella*, *Neisseria*, unclassified *Pasteurellaceae*, *Actinomyces, Veillonella*, *Rothia, Prevotella*, MiSeq sequencing	
176	M/F 3–4	*Leptotrichia* sp. oral clone FP036	Dental caries	Saliva	*P. melaninogenica*, *P. histicola*, *P. salivae*, *R. dentocariosa*, *Haemophilus, S. mutans*, *S. sanguinis*, *Neisseria, Gemella*, *Veillonella, Veillonella* sp. oral taxon 780, *A. odontolyticus*, *A. graevenitzii*, *Scardovia, F. periodonticum*, *Lactobacillus*, MiSeq sequencing	[69]
	M/F 3–4	*Leptotrichia* sp. oral clone FP036	Healthy	Saliva	*P. melaninogenica*, *P. histicola*, *P. salivae*, *R. dentocariosa*, *Veillonella, A. odontolyticus*, *A. graevenitzii*, *Veillonella* sp. oral taxon 780, *Haemophilus, F. periodonticum*, *Gemella, S. mutans*, *S. sanguinis*, *Neisseria*, MiSeq sequencing	
178	M/F 3–7	*L. buccalis*	Teeth with irreversible pulpitis	Cells	*C. rectus*, *G. morbillorum*, *T. denticola*, *F. nuc*. ssp. *polymorphum*, *C. ochracea*, *C. gingivalis*, *S. mitis*, *S. intermedius*, *S. gordonii*, checkerboard DNA–DNA hybridization	[70]
	M/F 3–7	*L. buccalis*	Teeth with pulp necrosis and apical periodontitis	Cells	*C. rectus*, *T. denticola*, *S. intermedius*, *S. mitis*, *S. oralis*, *S. gordonii*, *F. nuc*. ssp. *polymorphum*, *G. morbillorum*, *C. gingivalis*, *C. ochracea*, checkerboard DNA–DNA hybridization	
180	M/F 60–70	*L. buccalis*	ABL, control no bone loss	Subgingival plaque, guinea pigs	*L. acidophilus*, *E. corrodens*, *S. anginosus*, *S. sanguinis*, *S. mutans*, *S. oralis*, *E. saburreum*, *P. gingivalis*, *T. forsythia*, *F. nuc*. ssp. *vincentii*, *F. nuc*. ssp. *polymorphum*, *F. nuc*. ssp. *nucleatum*, *T. denticola*, *P. micra*, *P. intermedia*, *A. actinomycetemcomitans*, checkerboard DNA–DNA hybridization	[71]
181	M/F 18–70 ± 34	*L. buccalis*	Endodontic root canal infection	Tissue fluid	*P. melaninogenica*, *A. actinomycetemcomitans*, *P. gingivalis*, *F. nuc*. ssp. *nucleatum*, *F. nuc*. ssp. *vincentii*, *E. faecalis*, *A. israelii*, *A. naeslundii*, *A. gerencseriae*, *C. rectus*, *C. gracilis*, *N. mucosa*, *S. oralis*, *S. anginosus*, *S. intermedius*, *E. saburreum*, *V. parvula*, *C. ochracea*, checkerboard DNA–DNA hybridization	[72]
182	F 26–42	*L. buccalis*	Pregnant, postpartum, BOP	Supragingival sample	*N. mucosa*, *C. ochracea*, *C. sputigena*, *S. aureus*, *E. saburreum*, *F. nuc*. ssp. *naviforme*, *F. nuc*. ssp. *polymorphum*, *S. gordonii*, *S. anginosus*, *S. mutans*, *S. intermedius*, *S. sanguinis*, *S. oralis*, *V. parvula*, *P. micra*, *P. intermedia*, *P. melaninogenica*, *S. noxia*, checkerboard DNA–DNA hybridization	[73]
183	F 30	*L. buccalis*, *L. goodfellowii*	Heathy, dog bite, cellulitis, painful erythema, inflammation, R	Wound exudate (bacteremia)	*Capnocytophaga* spp., *C. perfringens*, culture, API rapid ID 32A, molecular identification	[74]
184	M/F 14–32	*L. buccalis*	Lip piercings – stainless steel-stud	Biofilms, absorbed fluid	*A. actinomycetemcomitans*, *V. parvula*, *T. denticola*, *P. micra*, *C. rectus*, *C. gracilis*, *C. showae*, *E. saburreum*, *P. melaninogenica*, *S. anginosus*, *S. oralis*, *S. mutans*, *S. intermedius*, *S. mitis*, *S. pneumoniae*, *S. constellatus*, *F. nuc*. ssp. (*naviforme*, *nucleatum, polymorphum)*, *F. periodonticum*, *C. gingivalis*, *C. sputigena*, *B. longum*, *G. vaginalis*, *S. aureus*, *S. anaerobius*, *S. haemolyticus*, *S. epidermidis*, *L. acidophilus*, *A. naeslundii*, *P. ginigvalis*, *P. aeruginosa*, checkerboard DNA–DNA hybridization	[75]
	M/F 14–32	*L. buccalis*	Lip piercings-titanium-stud	Biofilms, absorbed fluid	*A. actinomycetemcomitans, V. parvula, T. denticola, P. micra, C. rectus, C. showae*, *C. gingivalis*, *C. sputigena*, *P. melaninogenica*, *P. ginigvalis*, *E. saburreum*, *S. anginosus*, *S. mutans*, *S. intermedius*, *S. pneumoniae*, *S. mitis*, *S. oralis*, *C. gracilis*, *S. epidermidis*, *S. aureus*, *S. anaerobius*, *F. nuc*. ssp. (*naviforme*, *nucleatum, polymorphum)*, *F. periodonticum*, *S. haemolyticus*, checkerboard DNA–DNA hybridization	
	M/F 14–32	*L. buccalis*	Lip piercings – polypropylene-stud	Biofilms, absorbed fluid	*A. actinomycetemcomitans*, *P. melaninogenica*, *T. denticola*, *E. saburreum*, *S. mutans*, *S. anginosus*, *S. intermedius*, *S. mitis*, *S. oralis*, *F. periodonticum*, *F. nuc*. ssp. (*naviforme*, *nucleatum, polymorphum)*, *P. micra*, *C. rectus*, *C. gingivalis*, *C. gracilis*, *C. showae*, *V. parvula*, *G. vaginalis*, *S. aureus*, *S. anaerobius*, *S. epidermidis*, *S. haemolyticus*, checkerboard DNA–DNA hybridization	
	M/F 14–32	*L. buccalis*	Lip piercings-PTFE-stud	Biofilms, absorbed fluid	*A. actinomycetemcomitans, P. melaninogenica*, *S. anginosus*, *S. mutans*, *S. intermedius*, *S. oralis*, *E. saburreum*, *C. gracilis*, *C. rectus*, *C. showae*, *P. micra*, *V. parvula*, *G. vaginalis*, *F. periodonticum*, *F. nuc*. ssp. (*naviforme*, *nucleatum, polymorphum)*, *T. denticola*, *S. anaerobius*, *S. aureus*, *S. haemolyticus*, *S. epidermidis*, checkerboard DNA–DNA hybridization	
188	M/F 23–59	*L. buccalis*	Healthy	Saliva	*G. haemolysans*, *Veillonella* spp., *V. parvula*, *S. gordonii*, *S. mutans*, *S. oralis*, *S. thermophilus*, *S. termitidis*, virus, 314 chips sequencing	[76]
189	M/F 27–57	*L. buccalis*	Endodontic infection, swelling, sinus tract, exudates	Root canal, mouth	*E. faecium*, *E. faecalis*, *S. epidermidis*, *S. warneri*, *P. micra*, *H. pylori*, *E. saburreum*, checkerboard DNA–DNA hybridization	[77]
190	F 35	*L. buccalis*	Immunocompetent, pregnant, afebrile, AC, R, pregnancy loss (non-viable infant) Note: 1st case with acute chorioamnionitis	Amniotic fluid (bacteremia)	Culture, MALDI-TOF MS, bioMérieux Vitek MS, 16S rRNA gene sequencing	[78]
191	M/F 26–41	*L. buccalis*	Peri-implantitis	Peri-implant crevicular fluid	*P. aeruginosa*, *A. actinomycetemcomitans*, *F. periodonticum*, *A. israelii*, *E. coli*, *P. micra*, *S. anginosus*, *T. forsythia*, *S. aureus*, *S. haemolyticus*, *C. gracilis*, checkerboard DNA–DNA hybridization	[79]
192	M/F 40–60	*L. wadei*	DS, low gastric cancer risk	Antral gastric biopsies, Tumaco	*Veillonella*, *Staphylococcus, Haematobacter*, *Porphyromonas, Catonella*, *N. flavescens*, *Sphingomonadaceae, H. pylori*, *P. oris*, *Actinomyces*, TM7 genera *incertae sedis*, *S. oralis*, *C. gingivalis*, *Rothia, Flavobacterium*, 16S rRNA gene sequencing, HTS, PCA	[80]
	M/F 40–60	*L. wadei*	DS, high gastric cancer risk	Antral gastric biopsies, Túquerres	*H. pylori*, *Veillonella*, 16S rRNA gene sequencing, HTS, PCA	
	M/F 41–60		Cholelitiasis, non-*Opisthorchis felineus*, pancreatitis, hepatitis C virus	Aspirated bile	*Flectobacillus*, *Burkholderia, P. mexicana*, *Xanthobacter, A. lwoffii*, *A. johnsonii*, *L. brevis*, *J. psychrophilus*, *T. socranskii*, *T. amylovorum*, *V. dispar*, *R. aeria*, *Streptomyces, S. yabuuchiae*, *S. anginosus*, *R. caricis*, *H. influenza*, *J. lividum*, *B. uniformis*, *B. flexus*, *C. durum*, *S. xenophagum*, *M. mobilis*, *M. adhaesivum*, *S. equorum*, PCR, qPCT, HTS, PCA	
195	M/F 4–5	*L. wadei*, *L. hofstadii*	Halitosis, tongue	Saliva, tongue coating, mouth	*P. stomatis*, *E. sulci*, *E. saburreum*, *S. australis*, *Bacteroides*, TM7 genus *incertae sedis*, *Fusobacterium, Capnocytophaga*, *P. shahii*, *P. loesheii*, *P. catoniae*, *S. moorei*, *Actinomyces* spp., *A. graevenitzii*, *A. gerencseriae*, *S. infelix*, unclassified *Flavobacteriaceae* spp., 16S rRNA gene sequencing, HTS, qPCR, PCA	[81]
	M/F 4–5	*L. wadei*, *L. hofstadii*	Healthy, tongue	Saliva, tongue coating, mouth	*S. moorei*, *Actinomyces* spp., *P. stomatis*, *Capnocytophaga, A. graevenitzii*, *A. gerencseriae*, TM7 genus *incertae sedis*, *P. shahii*, *P. loesheii*, *P. catoniae*, unclassified *Flavobacteriaceae* sp., *Streptococcus* spp., *S. infelix*, *S. australis*, 16S rRNA gene sequencing, HTS, qPCA, PCA	
197	M 12–79	*L. hofstadii*	Tongue coating, halitosis	Saliva	*Granulicatella*, *Fusobacterium*, *Porphyromonas*, *Lautropia*, *Aggregatibacter*, *Haemophilus*, *Prevotella*, *Streptococcus*, *Treponema*, *Veillonella*, *Neisseria*, *Parvimonas*, *Rothia*, PCR, qPCR, T-RFLP, PCA	[82]
	F 12–79	*L. hofstadii*	Tongue coating, halitosis	Saliva	*Granulicatella*, *Fusobacterium*, *Streptococcus*, *Aggregatibacter*, *Parvimonas*, *Rothia*, *Veillonella*, *Haemophilus*, *Porphyromonas*, *Prevotella*, *Neisseria*, *Lautropia*, *Treponema*, PCR, qPCR, T-RFLP, PCA	
199	F 33–64	*L. hofstadii*	Periodontitis	SPPS	*Streptococcus* spp., Actinobacteria, Bacteroidetes sp. clone, TM7, *K. oralis*, *P. alactolyticus*, *Treponema* spp., *S. intermediu*, *S. intermedius*/*anginosus*, *S. parasanguinis*, *S. cristatus* spp., *S. anginosus*/*intermedius*/*constellatus*, *E. yurii*, *E. saphenum*, *E. brachy*, *S. satelles*, Synergistetes, HOMIM DNA microarray	[83]
	F 33–64	*L. hofstadii*	Periodontitis	GCF	*P. nigrescens*, *T. forsythia*, *Haemophilus* spp., *Peptostreptococcaceae* spp., *F. nuc*. ssp. *polymorphum*, *Actinomyces*, TM7, *C. rectus*/*concisus*, *C. concisus*, *D. pneumosintes*, Spirochaetes, Synergistetes, Bacteroidetes spp., HOMIM, DNA microarray	
201	US 6–8	*L. hofstadii*	Caries-active	Saliva	Bacteroidetes spp., *Lachnospiraceae, Selenomonas* spp., *Campylobacter, P. propionicum*, *Tannerella* spp., *S. infelix*, TM7 sp. clone, *P. micra*, *S. mutans*, *S. anginosus*, *Eubacterium, C. showae*, *C. matruchotii*, *C. sputigena*, *G. sanguinis*, *Prevotella, P. catoniae*, HOMIM 16S rRNA gene, microarray	[84]
202	F US	*L. goodfellowii*	Immunocompetent, foul odor, stillborn child, spontaneously expelled at 25 weeks of gestation, amniotic fluid, urinary tract infection, D	Gastric fluid, blood (bacteremia)	*E. coli*, culture, ANC Vitek 2, GC, 16S rRNA gene sequencing	[85]
203	M/F 3–6 ± 1.19	*L. shahii*	Healthy	Plaque, saliva, mouth	*S. oralis*, *C. leadbetteri*, *C. granulosa*, *N. mucosa*, *N. subflava*, *Ottowia* spp., *A. segnis*, *Porphyromonas* spp., urease activity, HTS	[86]
204	M 7	*L. trevisanii*, *L. buccalis*	Burkitt’s lymphoma, fever	Blood, ulceration, bacteremia, R	*P. canis*, *S. paucimobilis*, culture, VITEK 2 system, VITEK MS, Bruker Biotyper, MALDI-TOF MS, 16S rRNA gene sequencing	[87]
	M 37	*L. trevisanii*	Diffused large B-cell lymphoma, fever	Blood, bacteremia R	Culture, VITEK 2 system, VITEK MS, Bruker Biotyper, MALDI-TOF MS, 16S rRNA gene sequencing	
	M 65	*L. trevisanii*, *L. buccalis*	Plasmablastic lymphoma, fever	Blood, bacteremia mucositis, R	*S. paucimobilis*, culture, VITEK 2 system, VITEK MS, Bruker Biotyper, MALDI-TOF MS, 16S rRNA gene sequencing	
	F 34	*L. trevisanii*, *L. buccalis*	Double primary cancer (colon and gastric cancer), diarrhea	Blood, bacteremia R	*S. paucimobilis*, culture, VITEK 2 system, VITEK MS, Bruker Biotyper, MALDI-TOF MS, 16S rRNA gene sequencing	
	M 19	*L. trevisanii, L. buccalis*	Ewing sarcoma, fever	Blood, bacteremia ulceration, R	*S. paucimobilis*, culture, VITEK 2 system, VITEK MS, Bruker Biotyper, MALDI-TOF MS, 16S rRNA gene sequencing	
209	M/F 53.6 ± 14.6	*Leptotrichia* spp.	Healthy	Buccal scraping samples	*Streptococcus*, *Prevotella, Haemophilus*, *Fusobacterium, Actinomyces*, *Neisseria, Veillonella*, PCR, qPCR, HT-454 pyrosequencing	[88]
	M/F 48.2 ± 15.5	*Leptotrichia* spp.	Oral lichen planus patients, erosive	Buccal scraping samples	*Fusobacterium*, *Veillonella, Streptococcus*, *Prevotella, Haemophilus*, *Lautropia, Neisseria*, *Actinomyces*, PCR, HT-454 pyrosequencing	
	M/F 43.8 ± 14.1	*Leptotrichia* spp.	Oral lichen planus patients, non-erosive	Buccal scraping samples	*Streptococcus*, *Haemophilus, Fusobacterium*, *Actinomyces, Veillonella*, *Prevotella, Neisseria*, PCR, HT-454 pyrosequencing	
212	UK	*Leptotrichia* (OTU 11),	Healthy	Saliva, oral biofilm	*Neisseria* (OTU 5), *Streptococcus* (OTU 90), *Haemophilus* (OTU 3), *Rothia* (OTU 8, OTU 58), *Veillonella* (OTU 2, OTU 17, OTU 44), *Prevotella* (OTU 12, OTU 16, OTU 25), *Fusobacterium* (OTU 24), *C. albicans*, culture, CLSM, qPCR, PCA, HTS,	[89]
213	M 58 ± 2.3	*Leptotrichia* spp., *L. buccalis*	Normoglycemic non-smoker, periodontitis	Plaque, periodontitis	*Streptococcus*, *S. oralis*, *S. sanguinis*, *Neisseria, Selenomonas*, *Treponema, C. gracilis*, *C. matruchotii*, *F. nucleatum*, *G. sanguinis*, *G. adiacens*, PCR, FLX 16S pyrosequencing, PCA	[90]
	M 58 ± 6.6	*Leptotrichia* spp.	Hyperglycemic non-smoker, periodontitis, diabetics	Plaque, periodontitis	*Fusobacterium*, *Parvimonas, Peptostreptococcus*, *Peptostreptococcaceae* [XI] [G4] [XII] [G5], *Streptococcus, Veillonella*, *Acinetobacter, Tannerella*, *Pseudomonas, Terrahaemophilus*, *Lactobacillus, Lachnoanaerobaculum*, *F. alocis*, *Corynebacterium, Porphyromonas*, *Alloprevotella, Stenotrophomonas*, *Brevundimonas, Gemella*, *Microbacterium, Sphingomonas*, *Fretibacterium, Prevotella*, *Eubacterium, Atopobium*, *Neisseria, Haemophilus*, *Enterobacter, Bergeyella*, *Dialister, Treponema*, TM7, PCR, FLX 16S pyrosequencing, PCA	
	M 50 ± 9.8	*Leptotrichia* spp.	Normoglycemic smoker, periodontitis	Plaque, periodontitis	*Streptococcus*, *Stenotrophomonas,, Neisseria*, *Selenomonas, Alloprevotella*, *Brevundimonas, Pseudomonas*, *Acinetobacter, Treponema*, *Enterobacter, Bergeyella*, *Terrahaemophilus*, PCR, FLX 16S pyrosequencing, PCA	
	M 56 ± 6.3	*Leptotrichia* spp.	Hyperglycemic smoker periodontitis, diabetics	Plaque, periodontitis	*Fusobacterium*, *Parvimonas, Peptostreptococcus*, *Peptostreptococcaceae* [XI] [G4] [XII] [G5], *Gemella, Streptococcus*, *F. alocis*, *Veillonella*, TM7, *Terrahaemophilus, Corynebacterium*, *Lachnoanaerobaculum, Porphyromonas*, *Prevotella, Alloprevotella*, *Brevundimonas, Microbacterium*, *Sphingomonas, Acinetobacter*, *Stenotrophomonas, Lactobacillus*, *Fretibacterium, Dialister*, *Pseudomonas, Tannerella*, *Eubacterium, Atopobium*, *Hemophilus, Neisseria*, *Enterobacter, Bergeyella*, *Treponema*, PCR, FLX 16S pyrosequencing, PCA	
	M 40 ± 9.8	*Leptotrichia* spp.	Normoglycemic non-smoker	Plaque, without periodontitis	*Streptococcus*, *S. oralis*, *S. sanguinis*, *Neisseria, Selenomonas*, *Treponema, C. gracilis*, *C. matruchotii*, *F. nucleatum*, *G. sanguinis*, *G. adiacens*, PCR, FLX 16S pyrosequencing, PCA	
	M 49.2 ± 3.8	*Leptotrichia* spp., *L. goodfellowii*	Hyperglycemic non-smoker, diabetic	Plaque, without periodontitis	*Peptostreptococcus*, *Peptostreptococcaceae* [XI] [G4] [XII] [G5], *Gemella, G. sanguinis*, *Parvimonas, Fusobacterium*, *F. nucleatum*, *Streptococcus*, *S. oralis*, *S. sanguinis*, *Veillonella*, TM7, *Terrahaemophilus, Campylobacter*, *C. gracilis*, *F. alocis*, *Lactobacillus, Lachnoanaerobaculum*, *Fretibacterium, G. adiacens*, *Porphyromonas, Stenotrophomonas*, *Brevundimonas, Pseudomonas*, *Bergeyella, Corynebacterium*, *C. matruchotii*, *Stenotrophomonas, Alloprevotella*, *Microbacterium, Enterobacter*, *Treponema, Eubacterium*, *Acinetobacter, Prevotella*, *Atopobium, Tannerella*, *Dialister*, PCR, FLX 16S pyrosequencing, PCA	
	M 41.3 ± 6.3	*Leptotrichia* spp., *L. wadei*	Normoglycemic smoker	Plaque, without periodontitis	*Streptococcus, S. oralis, S. sanguinis*, *Neisseria, Selenomonas*, *Treponema, C. gracilis*, *F. nucleatum*, *Alloprevotella, Stenotrophomonas*, *C. matruchotii*, *G. sanguinis*, *Brevundimonas, Terrahaemophilus*, *Pseudomonas, Acinetobacter*, *G. adiacens*, *Enterobacter, Bergeyella*, PCR, FLX 16S pyrosequencing, PCA,	
220	M/F 40	*Leptotrichia*	Normal, smoker	Oral cavity swab samples	*Streptococcus*, *Veillonella, Gemella*, *Granulicatella, Neisseria*, *Haemophilus, Selenomonas*, *Fusobacterium, Lachnoanaerobaculum*, *Porphyromonas, Prevotella*, PCR, cloning, RFLP analysis, 16S rDNA sequencing, MOTHUR, AMOVA	[91]
	M/F 54	*Leptotrichia*	Oral potentially malignant disorder (OPMD), smoker, drinker	Oral cavity swab samples	*Streptococcus*, *Veillonella, Gemella*, *Granulicatella, Neisseria*, *Haemophilus, Selenomonas*, *Fusobacterium, Lachnoanaerobaculum*, *Porphyromonas, Prevotella*, PCR, cloning, RFLP analysis, 16S rDNA sequencing, MOTHUR, AMOVA	
	M/F 60	*Leptotrichia*	Oral cancer, smoker, drinker	Oral cavity swab samples	*Streptococcus*, *Veillonella, Gemella*, *Granulicatella, Neisseria*, *Haemophilus, Selenomonas*, *Fusobacterium, Porphyromonas*, *Lachnoanaerobaculum, Prevotella*, PCR, cloning, RFLP analysis, 16S rDNA sequencing, MOTHUR, AMOVA	
223	M 4.2 ± 0.5	*Leptotrichia* spp.	Healthy children without mite sensitization	Oropharyngeal swabs	Firmicutes, Proteobacteria, Bacteroidetes, Fusobacteria, Actinobacteria, *Streptococcus, Haemophilus*, *Prevotella*, *Moraxella* spp., *Porphyromonas, Fusobacterium*, *Parvimonas* PCR, MiSeq sequencing	[92]
	M 4.4 ± 0.4	*Leptotrichia* spp.	Mite-sensitized children with rhinitis	Oropharyngeal swabs	Firmicutes, Proteobacteria, Bacteroidetes, Fusobacteria, Actinobacteria, *Streptococcus, Haemophilus* spp., *Neisseria* spp., *Porphyromonas, Moraxella* spp., *Fusobacterium, Parvimonas* PCR, MiSeq sequencing	
	M 4.6 ± 0.3	*Leptotrichia* spp.	Mite-sensitized children with asthma	Oropharyngeal swabs	Firmicutes, Proteobacteria, Bacteroidetes, Fusobacteria, Actinobacteria,*Streptococcus, Haemophilus* spp., *Neisseria* spp., *Moraxella* species, *Porphyromonas, Fusobacterium*, *Parvimonas* PCR, MiSeq sequencing	
226	M/F 57	*L. wadei*, *L. wadei* (HOT-222)	Placebo group, exacerbation-prone severe asthma, lower respiratory tract infections	Oropharyngeal swabs	*L. orale*, *L. mirabilis*, *M. micronuciformis*, *O. sinus*, *S. moorei*, TM7 [G-1] sp., *G. sanguinis* (HOT-757), *Prevotella, P. melaninogenica* (HOT-469), *P. pallens* (HOT-714), *N. flavescens* (HOT-610), *G. adiacens* (HOT-534), *V. atypica* (HOT-524), *Streptococcus* spp., *S. mitis*/*pneumoniae* (HOT-677), *S. parasanguinis* (HOT-411), *S. salivarius*, *S. salivarius* (HOT-755), *F. periodonticum* (HOT-201), *A. graevenitzii* (HOT-866), *H. parainfluenzae* (HOT-718), PCR, 454 pyrosequencing, PCA	[93]
	M/F 48	*L. wadei*, *Leptotrichia* spp. (HOT-417 and HOT-225), *L. hofstadii* (HOT-224), *L. wadei* (HOT-222)	AZ responders, exacerbation-prone severe asthma, lower respiratory tract infections	Oropharyngeal swabs	*L. orale*, *L. mirabilis*, *M. micronuciformis*, *O. sinus*, *S. moorei*, TM7 [G-1] sp., *M. catarrhalis*, *H. influenza*, *H. parainfluenzae*, *H. parainfluenzae* (HOT-718), *A. graevenitzii* (HOT-866), *G. sanguinis* (HOT-757), *Streptococcus, S. parasanguinis* (HOT-411), *S. pneumonia*, *S. mitis*/*pneumoniae* (HOT-677), *S. salivarius*, *S. salivarius* (HOT-755), *G. adiacens* (HOT-534), *M. faucium*, *M. lipophilum*, *M. salivarium*, *Prevotella, P. melaninogenica* (HOT-469), *P. pallens* (HOT-714), *V. atypica* (HOT-524), *F. periodonticum* (HOT-201), *F. nucleatum* (HOT-200), *N. flavescens* (HOT-610), PCR, 454 pyrosequencing, PCA	
	M/F 48	*L. wadei*, *L. wadei* (HOT-222)	AZ non-responders, exacerbation-prone severe asthma, lower respiratory tract infections	Oropharyngeal swabs	*L. orale, L. mirabilis, M. micronuciformis, O. sinus, S. moorei*, TM7 [G-1] sp., *M. catarrhalis, H. influenza, H. parainfluenzae*, *H. parainfluenzae* (HOT-718), *A. graevenitzii* (HOT-866), *Streptococcus, S. parasanguinis* (HOT-411), *S. pneumonia*, *S. mitis*/*pneumoniae* (HOT-677), *S. salivarius*, *S. salivarius* (HOT-755), *V. atypica* (HOT-524), *N. flavescens* (HOT-610), *M. faucium*, *M. lipophilum*, *M. salivarium*, *Prevotella, P. melaninogenica* (HOT-469), *P. pallens* (HOT-714), *F. nucleatum* (HOT-200), *F. periodonticum* (HOT-201), *G. adiacens* (HOT-534), *G. sanguinis* (HOT-757), PCR, 454 pyrosequencing, PCA	
229–231	M/F 42.0 ± 14.6	*Leptotrichia* spp.	Normal, MBL	Saliva	*Veillonella*, *Haemophilus*, TM7, Tenericutes, *Neisseria*, *Oribacterium, Rothia*, *Selenomonas* [G-3], *Alloprevotella*, *Prevotella, Prevotella* [G-7], *Actinomyces, Lautropia*, *Granulicatella, Selenomonas*, *Capnocytophaga, Porphyromonas*, *Fusobaterium, Gemella*, *Streptococcus*, PCR, 16S MiSeq sequencing	[94]
	M/F 45.0 ± 14.1	*Leptotrichia* spp.	Moderate, MBL	Saliva	*Treponema*, TM7, Tenericutes, *Neisseria, Oribacterium*, *Selenomonas* [G-3], *Selenomonas, Porphyromonas*, *Fusobaterium Capnocytophaga*, *Lautropia, Granulicatella*, *Gemella, Alloprevotella*, *Prevotella, Rothia*, *Haemophilus, Veillonella*, *Prevotella* [G-7], *Actinomyces, Streptococcus*, *P. gingivalis*, *T. denticola*, PCR, 16S MiSeq sequencing	
	M/F 52.3 ± 15.9	*Leptotrichia* spp.	Severe, MBL	Saliva	*Treponema*, *TM7*, Tenericutes, *Streptococcus, Lautropia*, *Capnocytophaga, Neisseria*, *Oribacterium, Actinomyces*, *Prevotella* [G-7], *Porphyromonas, P. gingivalis*, *Prevotella, Alloprevotella*, *Selenomonas* [G-3], *Selenomonas, T. denticola*, *Fusobaterium, Granulicatella*, *Gemella, Haemophilus*, *Veillonella, Rothia*, PCR, 16S MiSeq sequencing	

A, adult; ABL, alveolar bone loss; AC, acute chorioamnionitis; ABCOPD, acute exacerbation of chronic obstructive pulmonary disease; AML, acute myelogenous leukemia; AMOVA, analysis of molecular variance; BALF, bronchoalveolar lavage fluid; BC, bladder cancer; BOP, bleeding on probing; BPES, black pigmented extrinsic stain; CAP, community-acquired pneumonia; CF, caries free; CLSM, confocal scanning laser microscopy; D, died; DI, diabetes; DO, days old; DS, dyspeptic symptoms; DU, duodenal ulcer; EG, erythematous gastropathy; F, females; GC, gas chromatographic; GCF, gingival crevicular fluid; GRD, gastroesophageal reflux disease; GU, gastric ulcer; H, healthy; HF, heart failure; HH, hiatal hernia; HNSCC, head and neck squamous-cell carcinoma; hrHPV, high-risk human papillomavirus; HSCT, hematopoietic stem-cell transplant; HTS, high-throughput sequencing; IMS, immunosuppression; M, male; MALDI-TOF MS, matrix assisted laser desorption ionization-time of flight mass spectrometry; MA, metabonomic analysis; MBL, marginal bone loss; MST, metagenome sequencing technology; MLD, mild liver dysfunction; mo, months; MY, months – years; NF, neutropenic fever; NHL, non-Hodgkin lymphoma; NTB, new tuberculosis; OPSCC, oropharyngeal squamous-cell carcinoma; PA, peritonsillar abscess; PBSB, peripheral blood smear blasts; PBSCT, peripheral blood stem-cell transplant; PCA, principal component analysis; PEDV, porcine epidemic diarrhea virus; PFGE, pulse field gel elctrophoresis; qPCR, real-time quantitative polymerase chain reaction; R, recovery; RD, respiratory distress; RE, reflux esophagitis; RSRTWI, redness-swelling – right tonsil-incision wound; RT, renal transplant; RTB, recurrent tuberculosis; SD, subsequently died; T, transgender; TFTB, treatment failure tuberculosis; UGIB, upper gastrointestinal bleeding; UK, unknown; US, unspecified; w, week.

In most cases, the cause of *Leptotrichia* infections has been *L. buccalis*. Since previous reviews [2,3], *Leptotrichia* species have been reported in >124 cases [4,7,16,18–69,87–93], whereby 30 cases involved *L. buccalis* [4,8,15,21,34,52,56,70–79,87], 24 cases *L. wadei* [4,20,24,34,37,42,48,56,67,80,81,90,93], 16 cases *L. trevisanii* [4,5,9,10,13,14,17,37,87], 14 cases *L. hofstadii* [34,40,49,56,81–84,93], 10 cases *L. goodfellowii* [4,11,12,21,56,74,85,87], eight cases *L. hongkongensis* [4,6,18,45,47,56], and five *L. shahii* [34,56,86]. *L. trevisanii* and *L. wadei* bacteremia are extremely rare; clinicians should consider these species in cases involving immunocompromised patients with oral lesions [4,5,13,17,87]. The aim of the present review is to update the knowledge on the genus *Leptotrichia* as given in previous reports, adding information published after 2008 [2,3].

## Taxonomy

*Leptotrichia* was recognized and described by van Leeuwenhoek in 1683, and the genus was established in 1879 by Trevisan [2,3]. *Leptotrichia* ferments carbohydrates, producing lactic acid as its major metabolic end product [2,3]. The primary habitat has been considered to be the oral cavity.

In Bergey’s Manual of 2005 [95] and based on comparative analysis of 16S rDNA sequences [31], the genus *Leptotrichia* is placed in the phylum Fusobacteria in the family II *Leptotrichiaceae* with *Leptotrichia* as the first genus. Other genera of this family include *Sebaldella*, *Sneathia*, and *Streptobacillus* [3,95].

The genus *Leptotrichia* comprises seven formally described species: *L. buccalis* is the type species of the genus, followed by *L. goodfellowii*, *L. hofstadii*, *L. hongkongensis*, *L. shahii*, *L. trevisanii*, and *L. wadei* (Figure 1) [1–3,6,96]. Their characteristics have been described in detail elsewhere [1,6,95] and will not be repeated here. *L. amnionii* is not validly published [2,97]. However, based on 16S rRNA gene sequences, *L. amnionii* was suggested to be transferred to the genus *Sneathia* [1,2], and recently, a strain with similar resemblances and features was characterized, renamed, and transferred to the genus *Sneathia* as *S. amnii* [98]. For this reason, *L. amnionii* will not be discussed in this review.

## Genomics

The whole genomes of 12 *Leptotrichia* species have been completely sequenced [99,100]. A short description of these species and their genomic features are given in Table 1. In addition, a large variety of 16S rRNA gene *Leptotrichia* nucleotide sequences exists in various databases (e.g. in HOMD; www.homd.org), NCBI GenBank, RDP, DNA data Bank of Japan (DDBJ), and other private databases. For instance, a survey from the NCBI GenBank showed that >4,800 *Leptotrichia* nucleotide sequences were registered and deposited as of 7 August 2017. The sequences came from material collected from humans, animals, fish, and ocean sediment. A representative phylogenetic tree based on 4,800 *Leptotrichia* sequences showing the diversity of the species aligned by ClustalW is given in Figure 1. The phylogenetic tree was generated by neighbor joining based on 500 bootstrap replicates and reconstructed with MEGA7 software (www.megasoftware.net).

## Conserved proteins of the phylum Fusobacteria

### Conserved signature inserts

Genome sequencing has provided insight into rich resources of molecular markers or signatures that are specific for different groups of bacteria. These novel molecular markers can be used to demarcate diverse bacterial taxa. An example is conserved signature inserts (CSIs) or deletions (i.e. indels) in protein sequences [100].

Members of the family *Leptotrichiaceae* are easily distinguished based on concatenated sequences for conserved proteins. Comparative analysis of Fusobacteria identified CSIs in proteins involved in a broad range of functions specific for the phylum. Some of these CSIs important proteins are uniquely present in the protein homologs of all sequenced members of Fusobacteria and thereby provide potential molecular markers for this phylum, which includes the family *Leptotrichiacaeae*. Further, it has been suggested that these specific CSIs provide evidence that could be used as novel tools for identifying and distinguishing members of the families *Fusobacteriaceae* and *Leptotrichiaceae* and other bacteria [100]. The gene sequences for many of the proteins containing these CSIs are highly conserved and based upon the conserved regions of the genes/proteins, for which PCR primers can be designed.

### Clinical importance of *Leptotrichia* species

Eribe and Olsen [2,3] reported previously that the clinical importance of *Leptotrichia* species remains unclear due to difficulties in isolation and identification of the organisms [2,3,70]. Recently, with modern molecular techniques and more awareness, more light has been shed on *Leptotrichia* species and their involvement in a variety of diseases. *Leptotrichia* species commonly colonize the mucous membrane of humans and animals, and are significant constituents of the microbiota of the human oral cavity, playing an important role in many diseases [2,3,100]. Table 2, a continuation of previous Table 1 [2], depicts 176 cases of *Leptotrichia* species presented in the current review. It shows where *Leptotrichia* species were isolated, the various sources they came from, which *Leptotrichia* species were detected, the polymicrobial species they are associated with, as well as their frequencies. As can be seen, *Leptotrichia* species are commonly present in the human and animal gastrointestinal tract, in the periurethral region, and in the genitalia of women [1–3,21,54,97].

In a previous review [3], it was concluded that *Leptotrichia* species were isolated and recovered from various sources, including patients who had gingivitis, necrotizing ulcerative gingivitis, adult/juvenile periodontitis, ‘refractory periodontitis’, Vincent’s angina, noma, acute appendicitis, bacterial vaginosis, aortic aneurysms, cellulitis, phagedenic chancroid, saplpingitis, neutropenia, human immunodeficiency virus (HIV), leukemia, endocarditis, and human and animal infections [2,97]. It was suggested that *Leptotrichia* species are opportunistic pathogens. Current documentation and a review of the literature support this view.

## Brief additional clinical information on *Leptotrichia* species

### L. buccalis

Recently, *L. buccalis* has been isolated from irreversible pulpitis, pulp necrosis, apical periodontitis [70], and dental plaques of both humans and guinea pigs with alveolar bone loss (Table 2) [21,56,71,90]. It has also been recovered from root canals of patients with or without other oral diseases, tissue fluids and subgingival plaque samples, and exudate with cellulitis after a dog bite (Table 2) [8,52,72–74,77,90]. Furthermore, it has been recovered from the blood and amniotic fluid of a female patient and from the amniotic fluid of an afebrile pregnant woman with acute chorioamnionitis [4,78] (Table 2). It has also been detected in saliva, on the mucosal surface of patients with removable partial dentures, in peri-implant crevicular fluids [34,76,79], and in biofilms (Table 2) [75]. In addition, *L. buccalis* was isolated from the blood of an elderly woman who suffered from moderate normocytic anemia, acute myelogenous leukemia, and mucositis (Table 2) [15,87].

### L. goodfellowii

*L. goodfellowii* has been isolated from oral swabs of guinea pigs [21] and the gastric fluid of patients who suffered spontaneous stillborn child expulsion [85]. It has also been isolated from the blood of an amniotic fluid patient with a wound and respiratory difficulties [4], from a wound exudate of a healthy person with cellulitis after a dog bite [74], from saliva, plaque, and the mucosal surface of caries-active patients and diabetic smokers [56,90], and from the blood of patients with heart failure, diabetes, bladder cancer, pulmonary edema, and bronchopneumonia [11]. *L. goodfellowii* has been recovered from an immunocompetent endocarditis patient with bioprosthetic pulmonic valve and an aortic valve homograft suffering from fever and chronic night sweats (diaphoretic) (Table 2) [12].

### L. hofstadii

*L. hofstadii* has been isolated from subgingival samples and gingival crevicular fluid of periodontitis patients [83], saliva, biofilm from caries [49,65], the mucosal surface of patients with removable partial dentures, and root canals of patients with or without disease [34,56,84], tumor tissue [40], and tongue coatings of halitosis patients (Table 2) [81,82].

### L. hongkongensis

*L. hongkongensis* has been isolated from the blood of metastatic breast carcinoma (MBC) patients [6], the blood and amniotic fluid of a patient with a wound and respiratory difficulties [4], plaque from dental caries [45,47,56], saliva from pancreatic cancer patients and black pigmented stain caries patients (Table 2) [18,63].

### L. shahii

*L. shahii* has been recovered from the saliva and plaque of patients with active caries and the mucosal surface of patients with removable partial dentures (Table 2) [34,56,86].

### L. trevisanii

*L. trevisanii* has been cultured from the blood of an immunocompetent patient, dental plaque and stool of patients with stomatitis, neutropenia, mucositis, peritonsillar abscess, blood progenitor-cell transplantation, catheter-related bloodstream infection, acute myelogenous leukemia, and redness and swelling in a tonsil incision wound [5]. It has also been associated with mild liver dysfunction, normal renal function [5], multiple myeloma, non-Hodgkin lymphoma (NHL), diffuse large B-cell lymphoma, post-transplant aplasia, neutropenic fever, myelodysplastic syndrome, mandibular tumor, esophageal carcinoma, and the wound and amniotic fluid of a patient with respiratory difficulties [4,5,9,10,13,14,17,37,87].

### L. wadei

*L. wadei* has been isolated from bronchoalveolar lavage fluid of a patient with leukocytosis, hypoxemia, and dyspnea [24] and from the blood and amniotic fluid of a patient with a wound and respiratory difficulties (Table 2) [4]. Saliva, plaque, and the oral mucosal surface of caries patients [34,37,56,67] and the oral cavity and biofilms from oral epithelial cells of a patient with new-onset rheumatoid arthritis [20,48] all contained *L. wadei*. Patient material from tongue plaque, saliva, and the tongue coating of malodor and halitosis patients [42,81] was isolated with *L. wadei* present. This bacterium was even isolated from the antral gastric biopsy of a dyspeptic patient [80], smokers’ plaque [90], and oropharyngeal samples (Table 2) [93].

### Unspecified *Leptotrichia* species

*Leptotrichia* species have been recovered from the blood of patients with liver abscesses, mucositis, neutropenic sepsis, diabetes, respiratory distress, community-acquired pneumonia (CAP), bilateral lung crackles, mild anemia, and vasculitis (Table 2) [7,22,33–35]. They were also recovered from oral plaque of guinea pigs [21] and feces of piglets [54], dental plaque from healthy individuals, plaque and saliva from patients with various types of caries, gingivitis, chronic periodontitis, and peri-implantitis [23,25–27,34,35,37,38,44,45,47,49,52,59,60,62,66–69,91,94], decayed tooth surfaces and discordant caries from intact enamel surfaces [53]. *Leptotrichia* species were also isolated from bronchoalveolar lavage fluid, and patients with leukocytosis, hypoxemia, and dyspnea [24]. Further, *Leptotrichia* species were recovered from healthy patients with oral cancer, premalignant oral lesion [18,28,33,56,91], edentulous infants [29], human vaginal fluid of sexually active and inactive individuals [30,32], HIV-seropositive and -seronegative patients [46], pancreatic cancer patients [18,66], black pigmented stain caries patients [63], and patients with halitosis (Table 2) [42,65,81,82]. Besides, *Leptotrichia* species were isolated from the blood [4,5,22,74], the amniotic fluid of a patient with a wound and respiratory difficulties [4], breast milk of obese women with gestational diabetes and normal weight [36], oral samples of a patient with new-onset rheumatoid arthritis [20], oral lichen planus patients [88], and even from fermenting Lees liquor [39]. *Leptotrichia* species were equally isolated from the blood and gastric fluid of patients with coronary artery disease (CAD), candidal esophagitis, chronic kidney disease, diabetic, duodenal ulcer, erythematous gastropathy, gastroesophageal reflux disease, gastric ulcer, hiatal hernia, reflux esophagitis, upper gastrointestinal bleeding, renal transplant, and sarcoidosis (Table 2) [16]. Also, *Leptotrichia* species were isolated from tumor tissues and sputum of patients with tuberculosis, acute exacerbation of chronic obstructive pulmonary disease, and feces of piglets with porcine epidemic diarrhea virus [40,41,43,50,51]. They were also detected in patient material from tongue plaque with malodor [42], biofilms of caries, oral epithelial cells [48,49], vaginal swabs with high-risk human papillomavirus, and from HIV-positive and -negative subjects [55]. The guts of herbivorous, carnivorous, and omnivorous fish [58], tumor tissues and saliva of patients with head and neck squamous-cell carcinoma human papillomavirus (HPV), oropharyngeal squamous-cell carcinoma HPV, and oral cavity squamous-cell carcinoma HPV [19] all contained *Leptotrichia* species. They were also isolated from the bile aspirate of fish with cholelithiasis (gallstone diseases) and *Opisthorchis felineus* (fish-borne liver fluke infections), in pancreatitis and hepatitis C [61], and in saliva from a Behçet’s disease patient [64]. Wu et al. [57] reported recovery of *Leptotrichia* species, together with *Veillonella parvula* and *Peptostreptococcus* species in low amounts in cigarette smokers’ mouthwash (Table 2) [57,90,91]. Also, human skin emanation samples and oropharyngeal samples of mite-food-sensitized children with rhinitis and asthma were found to contain *Leptotrichia* species [31,92].

### Pathogenicity of *Leptotrichia*

The genus *Leptotrichia* consists of slow-growing, non-motile facultative anaerobic/anaerobic Gram-negative rods that reside in the oral cavity and the genitourinary and intestinal tract [1]. *Leptotrichia* species were traditionally considered non-pathogenic but have recently been considered as opportunistic causes of human disease [2,3,78]. Previously, Eribe and Olsen [2] described a myriad of pathological conditions associated with *Leptotrichia*, including appendicitis, pneumonia, mucositis, and sepsis [2,78]. The concept that Leptotrichia infections are opportunistic is further supported in the current review. *Leptotrichia* species, primarily oral commensals, have been associated with infections, particularly in immunocompromised hosts (Table 2) [4,9,13–17,24,30,32,46,55,74,78,97], but occasionally in immunocompetent persons [5,11,12,24,33,60,74,78,85].

The cell surface of leptotrichia has protruding structures presumed fitted for adhesion [2,3]. Like any other Gram-negative rod that possesses lipopolysaccharide (LPS, endotoxin), *Leptotrichia* displays O-antigen linked to lipid-A. The latter may cause hemorrhage, fever, tumor necrosis, fatal shock, and septicemia [4–7,9,10,12–15,17,24,33,40,85,87] and may even lead to abortion, as observed in infection associated with *L. goodfellowii* [85]. The virulence of *L. buccalis* was demonstrated experimentally in a rabbit model [2,3]. When Leptotrichia endotoxin was compared to Escherichia coli endotoxin in terms of a lethal dose for 50% survival, febrile response, and leukopenia, Leptotrichia endotoxin was 10–20% as active on a weight basis. In the same test, the endotoxin from *L. buccalis* proved more potent than *Salmonella* endotoxin.

### *Leptotrichia* and proinflammatory mediators

It is known that the systemic release of endotoxin and proinflammatory mediators from infected host tissue can contribute directly or indirectly to the sepsis syndrome associated with *Leptotrichia* [2,3,7]. Once activated, the immune system is hard to switch off, and sometimes it gets out of control, causing damage to other parts of the body. This ‘self-inflicted’ damage acts as trigger for various disease conditions [101]. Many types of Gram-negative bacteria secrete LPS that stimulates the immune system. A study by Langfeldt et al. [48] found that *Leptotrichia* was able to trigger the transcription level of proinflammatory interleukin (IL)-1β, IL-6, IL-8, and IL-10 in epithelial cells [48]. This suggests that *Leptotrichia* may play a key role during the transition from health to disease [54]. IL-1β modulates human cell differentiation, proliferation, and apoptosis, which regulate the release of other proinflammatory cytokines such as IL-6 and IL-8 [48]. In addition, IL-6 and IL-8 have the capacity to attract granulocytes and lymphocytes, thereby inducing the host cellular immune response. In contrast, IL-10 is designated as an anti-inflammatory mediator that prohibits excessive immune response by suppressing pro-inflammatory cytokine production and the antigen-presenting capacity of monocytes, macrophages, and dendritic cells [48]. Both pathogenic and commensal bacteria interfere with early host cell signalling for survival or promote bacterial infection by decreasing pro-inflammatory responses [48]. In an *in vitro* multispecies biofilm model with or without major periodontal pathogens, it was documented that such biofilms can upregulate IL-8 expression in gingival epithelial cells. The presence of the ‘red-complex’ species (*Porphyromonas gingivalis*, *Tannerella forsythia*, and *Treponema denticola*) resulted in even greater upregulation [48]. The data strongly argued that *Leptotrichia* may be crucially involved in the ‘fine-tune’ regulation of epithelial immune response to obtain homeostasis or propagate inflammatory response [48]. Jang et al. [102] reported that *L. wadei*, *Fusobacterium nucleatum*, and *Campylobacter gracilis* when co-cultured with human gingival fibroblasts highly upregulated the expression of antimicrobial chemokine peptides and the proinflammatory mediators IL-6 and IL-8, whereas the red-complex bacteria stimulated low levels or often suppressed expression of these factors [102].

New-onset patients with chronic rheumatoid arthritis harbored high levels of several pathogens, including *Gemella morbillorum*, *Propionibacterium acnes*, *Streptococcus gordonii*, and *L. buccalis*. This indicated that *L. buccalis* can be more specifically associated with multiple disease activity than so far realized [20,52]. Irrespective of periodontal disease status, the *Leptotrichia* OTU 87 (*L. wadei*) clone and *Prevotella* OTU 60 (*P. intermedia*) clone were the only clones observed in increased amount in patients with new-onset rheumatoid arthritis but were absent in healthy controls [20].

### *Leptotrichia* species in bacteremia

Thirty-one cases of bacteremia and four cases of wound infections associated with *Leptotrichia* species have been reported (Table 2). Bacteremia caused by *Leptotrichia* species were found among neutropenic patients with various forms of predisposing diseases such as bone-marrow transplants, infective endocarditis, and sepsis associated with mucositis. The latter served as an oral or orodental portal of entry [2,3,22]. In fact, neutropenic fever coupled with mucositis is an established predisposing factor for development of sepsis by *Leptotrichia* species [4,7,87]. Peripheral blood stem-cell transplant patients (PBSCT) with fever due to mucosal disruptions and lesions have a portal of entry for bacteria that causes bacteremia [5,9,22,33]. Mucositis, esophageal ulcer, or diverticulitis are possible risk factors for infected patients [7,9,13,15–17,33,85]. *L. trevisanii* was involved in 15 incidences of bacteremia. Eight cases each also involved *Leptotrichia* species and *L. buccalis*, six *L. goodfellowii*, three *L. wadei*, two *L. hongkongensis*, and one with *L. shahii* (Table 2) [4–6,9–15,17,21,22,33,42,74,78,85–88]. In cases involving *L. trevisanii*, seven were also associated with neutropenic fever [5,13,14,17,87], while five were associated with PBSCT [9,10], four had acute myelogenous leukemia (AML) [5,7 9,10,15] and multiple myelomas (MM) [4,9,13], two had stomatitis [10,14], three had NHL [9,87], and one had a catheter-related bloodstream infection [17]. It is worth mentioning that *L. goodfellowii* has previously been associated with endocarditis. *L. goodfellowii* isolated from immunocompetent patients was found to be a pathogenic agent when associated with bacteremia [11,12,33,74,85]. Lim et al. [11] therefore hypothesized that *L. goodfellowii* could be secondary to pneumonia, as there was no other causative factor leading to bacteremia in their patient. In one of three cases, *L. goodfellowii* was even associated with a stillborn child, spontaneously expelled after 25 weeks of gestation [4]. In three cases of *L. hongkongensis* bacteremia, one case was associated with amniotic fluid, fever, and MBC [6]. *L. wadei* bacteremia was detected in wounds and amniotic fluid [4].

Thus, recent reports have proven the pathogenicity of *Leptotrichia* species. Inappropriate clinical situations can affect the protective function of the indigenous bacterial flora, which can lead to disruption by broad-spectrum antibiotic therapy [2–4,12,69,103], resulting in infection. Likewise, enhanced *Leptotrichia* proliferation and tissue invasion can culminate in bloodstream invasion and dissemination [2,3]. This occurs frequently when the patient’s immune system is comprised with *Leptotrichia* species such as with cases involving *L. buccalis*, *L. trevisanii*, *L. wadei*, and *L. goodfellowii*. These species have been reported to act as opportunistic pathogens responsible for bloodstream infections in immunocompromised patients [2,4,5,15,17,33,74,85,87,103].

*L. buccalis* has been associated with chorioamnionitis and child loss during pregnancy [78]. The authors suggested that the development of chorioamnionitis was a result of hematogenous spread arising from the oral cavity [78]. Unique to bacteremia from other *Leptotrichia* species, *L. goodfellowii* showed an association with bacteremia secondary to endocarditis [11,12]. In contrast to previously reported cases of *Leptotrichia* bacteremia, the patient in this report was immunocompetent and had no history of endocarditis. For the first time, a case of *L. goodfellowii* bacteremia was recently reported in a Korean patient [11]. It is noteworthy that in a 62-month retrospective survey of 4,857 episodes of anaerobic bacteremia, *Leptotrichia* species were identified as the causative pathogens in 7.3% of cases [12,22].

### *Leptotrichia* species in cancers

A few *Leptotrichia* species were related to 88 incidences of various cancers [4–7,9–11,13–15,17–19,22,28,33,40,57,60,61,66,74,80,87,91], of which 43 cases had neutropenia, sepsis, and fever [4–7,9,10,12–15,17,22,24,33,87], 14 had transplant issues [4,9,10,13,16,17,22,33], 14 mucositis [4,7,9,13–15,17,22,87], 12 various lesions (6, 11, 27, 32, 37, 44, 56 64, 99), and five pneumonia [5,9,11,24,33]. The suspected port of *Leptotrichia* entry included mucositis, abscesses, wound infections, gingivitis, diverticulitis, peritonitis, neutropenic sepsis, and ulcers (Table 2).

In an examination of the relationship of the oral microbiota with subsequent risk of pancreatic cancer in a large nested case-control study, the authors reported that the carriage of oral pathogens, *P. gingivalis* and *Aggregatibacter actinomycetemcomitans*, was associated with a higher risk of pancreatic cancer [66]. They also found that a greater abundance of the phylum Fusobacteria was associated with decreased pancreatic cancer risk as well as its genus *Leptotrichia* [66]. Their finding was inconsistent with a recent cross-sectional study of eight patients, which found higher abundances of *Leptotrichia* and *Porphyromonas* in the saliva of pancreatic cancer patients compared to controls and those with other diseases, including non-cancerous pancreatic disease [18]. Torre et al. [18] concluded that the *Leptotrichia* and *Porphyromonas* ratio may serve as a potential pancreatic cancer biomarker. Based on their findings, pancreatic cancer may be detected at early stages by sampling individuals’ saliva and looking at the ratios of *Leptotrichia* and *Porphyromonas*.

### *Leptotrichia* in dental caries

Among the many microbial species residing in oral biofilms (plaque) at the tooth surface [104], *mutans* streptococci have long been recognized as primary contributors in the etiology of dental caries [104]. The pathogenicity of organisms such as *Streptococcus mutans* and *S. sobrinus* is attributable in part to (i) the capacity of these species to produce extracellular glucan(s) from dietary sucrose that facilitate microbial adherence to the tooth surface, and (ii) the fermentation of sucrose to lactic acid – the causative agent in the demineralization of tooth enamel [104]. There is supporting evidence that the genus *Leptotrichia* is highly saccharolytic [1–3,11,104–106], implying that it ferments a wide variety of mono- and disaccharides to lactic acid similar to *S. mutans*. This property may implicate the participation of *Leptotrichia* species in tooth decay [1–3,11].

## Association between *Leptotrichia* and halitosis

*Leptotrichi*a has also been associated with halitosis (oral malodor) [42,65,81,82]. Most of the species within the core microbiome of the tongue-coating biofilm are Gram-negative anaerobic bacteria that are adaptable to the tongue-coating environment (Table 2) [81]. Malodor is foul-smelling breath from the oral cavity in humans [42]. Most malodor originates from the host’s tongue plaque and is without any clear signs of disease, which is defined as physiologic oral malodor [42]. Malodorants are produced by the tongue plaque resident on the large surface area of the tongue. Some bacteria inside tongue plaque can produce amino acids and peptide by-products as well as food debris to putrefy, thus producing malodorants [42]. The unpleasant oral odor results from volatile sulfur compounds (VSCs), including hydrogen sulphide (H_2_S), methyl mercaptan (CH_3_SH), other thiols, and dimethyl sulphide ((CH_3_)_2_S) involved and associated with halitosis [42]. Of the three major VSCs involved in oral malodor, (CH_3_)_2_S is the main contributor to halitosis [81], whereas CH_3_SH is more pathogenic than H_2_S and is associated with periodontal disease [81]. It has been inferred that the reason for halitosis might be cooperative polymicrobial activity, which includes *Leptotrichia* species interactions rather than the effect of a single pathogen [81]. There is also evidence supporting that *Leptotrichia* species are present in increased abundances in people with oral malodor, despite a lack of H_2_S production [81,82]. Yang et al. reported that *L. wadei* was positively correlated with H_2_S concentrations [42] and concluded that *Leptotrichia* spp. and *Prevotella* spp. were found to be strongly associated with oral malodour [42], although direct proof of production was not provided. This bacterium was detected in relatively high abundance in all the halitosis tongue-coating samples and was inferred to be involved in halitosis [81,82], likewise *L. hofstadii* in some subjects [81,82]. Bacteria such as *Peptostreptococcus stomatis* and *Prevotella shahii* isolated from tongue coatings of diseased persons together with *L. wadei* were also suggested to be candidate halitosis pathogens [81] (Table 2).

### *Leptotrichia* in co-existence with other microbes

The human oral cavity has an indigenous microbiota known to include a robust community of microorganisms. *Leptotrichia* species are present in the salivary milieu and coexist with virus/bacteriophages in this environment, together with other microbes, for example *Veillonella* [76]. Their interrelationships remain elusive. *Leptotrichia*, *Clostridium*, and *Citrobacter* were found as the most abundant bacteria in the herbivorous fish gut [58]. Previous studies have reported that *Clostridium*, *Citrobacter, Leptotrichia*, *Bacillus*, and *Enterobacter* are important cellulose-degrading bacteria in herbivorous fish [58]. It was suggested that these bacterial species might play significant roles in their host’s digestive system. Herbivorous fish harbored abundant cellulose-degrading bacteria, including *Clostridium*, *Citrobacter*, and *Leptotrichia* (Table 2) [58]. *L. hofstadii* was considered and reported as a potential biomarker for dental caries in association with *Campylobacter showae* and *Parvimonas micra* [69,84]. *Leptotrichia* species were found together with *Fusobacterium* and *Campylobacter* species in patients with colorectal carcinoma. This polymicrobial signature was associated with overrepresentation of numerous host genes, including the gene for encoding the proinflammatory chemokine IL-8 [40].

*Leptotrichia* species were reported in close association with fungi, including species of *Saccharomyces*, *Aspergillus, Zygosaccharomyces*, *Pichia, Saccharomycopsis*, *Talaromyces, Eurotium*, *Fomitopsis, Trichosporon*, *Candida albicans*, *C. parapsilosis*, and *C. tropicalis*, and other species from liquor [39], gastric fluid [16], the saliva of HIV patients [46], sputum [50], blood, and saliva [60] (Table 2). The importance of these associations remains unknown. *Leptotrichia* species, together with *Delftia* species and *Actinobacteria* species, were significantly correlated with individuals attacked by malaria mosquitoes [31]. *Leptotrichia* species, *L. wadei*, and *Streptococcus* species were isolated together with *C. albicans* from dental plaque samples of patients with or without rampant caries [67,89]. The authors postulated that these pathogenic species and dysbiosis of the oral microbial community might have contributed to the pathogenesis of rampant caries in their patient. *Leptotrichia* spp. and *Lautropia* spp. were found to increase significantly in oral lichen planus (OLP) patients [88]. The argument for this was that as OLP is an immune-related disease, the elevated colonization of these bacteria might be related to the local immune dysfunction of OLP, which again suggested that OLP is associated with dysbiosis of the oral microbiome [88]. Kawanami et al. [24] suggested that in a severe pneumonia patient, isolated *L. wadei* and other *Leptotrichia* species, together with mixed oral bacteria (*Enterococcus faecalis*, *E. casseliflavus*, *Veillonella parvula*, *V. atypica*, *V. dispar*, *Prevotella nanceiensis*, *Streptococcus* spp. clones, *Delftia* sp. clone, *Lactobacillus* sp. clone, *Syntrophococcus* sp. clone, *Clostridium* sp. clone, and *Actinomyces* sp. clone), played important roles (Table 2) [24].

### Identification of *Leptotrichia* species

Identification of *Leptotrichia* species can be problematic in terms of culturing because some strains are strictly anaerobic or facultative anaerobic, while others prefer growth under the influence of CO_2_. *Leptotrichia* species usually stain Gram-negative, but fresh cells may be Gram-positive. Old cells may even stain both ways, leading to misclassification.

Due to the insufficiency of databases, identification of *Leptotrichia* species by conventional biochemical assays may be difficult and challenging, since most species are not recorded in databases. Most databases contain only one or two species known as *L. buccalis* or *Leptotrichia* species. Schrimsher et al. [9] reported cases of misidentification of *L. trevisanii* sepsis where all the isolates were unidentified by biochemical tests. One of the isolates was misidentified as *Sphingomonas paucimobilis* [9] and another as *Clostridium acetobutylicum* [13]. A report from Lim et al. [11] showed misidentification of *L. trevisanii* as *Capnocytophaga* spp. and *L. buccalis* by the Vitek 2 system [11], or as unidentified using this system. In addition, the MALDI-TOF MS system may struggle in the identification of *Leptotrichia* species [11]. The VITEK MS database has no known *Leptotrichia* species, making their identification impossible and underestimated. Lim et al. [11], however, reported that the Bruker Biotyper System (Bruker Daltonics, Billerica, MA), which contains some *Leptotrichia* species in their database, gave successful identification [11]. It is of general interest that more database development and strain accumulation are made to enable the precise identification of *Leptotrichia* species [11]. To avoid misclassification of *Leptotrichia* species, application of 16S rRNA gene identification is recommended because of its reliability and feasability. HOMD with its large amount of genetic data from oral bacteria is probably the most reliable database to use.

### Antimicrobial agents toward *Leptotrichia*

*Leptotrichia* species are susceptible to many antimicrobial agents such as penicillin, ampicillin, oxacillin, cephalothin, cefoxitin, cefotaxime, amoxicillin/sulbactam, ampicillin/sulbactam, amoxicillin/clavulanate, clindamycin, metronidazole, rifampicin, tetracycline, imipenem, and chloramphenicol. Strains have developed resistance to erythromycin, gentamycin, kanamycin, vancomycin, ciprofloxacin, tobramycin, amikacin, fluoroquinolones, and aminoglycosides [2,11,70]. Some strains have been treated successfully while others have not with these antibiotics. *L. goodfellowii* bacteremia has been successfully treated with piperacillin/tazobactam, ceftriaxone/metronidazole, or amoxicillin/clavulanate, clindamycin, vancomycin, gentamycin, and imipenem [11,74]. *L. goodfellowii* was found resistant to tobramycin, amikacin, and ciprofloxacin [74]. With antimicrobial susceptibility testing, prompt and adequate selection of antibiotics could be sufficient for treatment of *L. goodfellowii* bacteremia [11]. Antibiotic treatment with piperacillin/tazobactam, moxifloxacin, piperacillin, erythromycin, levofloxacin, gentamycin, amikacin, and chloramphenicol was unsuccessful toward *L. trevisanii* [13,14,17] and successful with meropenem [14,17], penicillin, amoxicillin, amoxicillin/clavulanate, cefoxitin, imipenem, clindamycin, tetracycline, metronidazole [13,14], cefotaxime, ceftazidime, piperacillin/tazobactam, and tigercycline [14]. Severe pneumonia caused by *L. wadei* was successfully treated with imipenem/cilastin, minocycline, sulfametoxazole/trimethroprim, and clindamycin but not with cefcapene pivoxil or levofloxacin [24].

### Clustered regularly interspaced short palindromic repeats in *Leptotrichia*

There is evidence that almost all *Archaea* and about half of *Bacteria* possess clustered regularly interspaced short palindromic repeats (CRISPRs). These are segments containing short repetitions of base sequences. The unique sequences between the repeats match the DNA of the virus preying on the bacterium. CRISPRs are part of the bacterial immune system. CRISPR-associated proteins (*Cas*) are adaptive immune systems for *Archaea* and *Bacteria* defending microbes against foreign genetic elements (e.g. virus) *via* DNA or RNA-DNA interference [107,108]. Most Cas proteins are grouped into two functional modules: (i) the adaptation module, which delivers genetic materials into CRISPR arrays generating CRISPR RNAs (crRNAs); and (ii) the effector module, which is guided by crRNA that targets and cleaves invading nucleic acids [107]. Up-to-date characterized CRISPR-Cas systems consist of Cas1 and Cas2, which are exclusively involved in spacer acquisition [107]. C2c2 is the sole effector protein that uses a crRNA guide to achieve interference, targeting RNA [107]. Targeting C2c2 to mRNA prevents gene expression [107], suggesting that CRISPR-Cas systems and C2c2 can be used for development of a new molecular RNA-targeting tools [107], including tools for *Leptotrichiaceae*. C2c2 from *L. shahii* was documented to provide interference against RNA phage [108].

## References

[1] Eribe ERK, Paster BJ, Caugant DA, et al Genetic diversity of *Leptotrichia* and description of *Leptotrichia goodfellowii* sp. nov., *Leptotrichia hofstadii* sp. nov., *Leptotrichia shahii* sp. nov. and *Leptotrichia wadei* sp. nov. Int J Syst Evol Microbiol. 2004;54:583–31. 15023979 10.1099/ijs.0.02819-0

[2] Eribe ERK, Olsen I. *Leptotrichia* species in human infections. Anaerobe. 2008;14:131–137. 18539056 10.1016/j.anaerobe.2008.04.004

[3] Eribe ERK, Olsen I *Leptotrichia* and *Leptotrichia*-like organisms In: Liu D, editor. Molecular detection of human bacterial pathogens. Section III. *Baceroidetes, Chlamydiae*, and *Fusobacteria*, Chapter 49. Boca Raton, London, New York: CRC Press: Taylor & Francis Group; 2011 p. 555–566.

[4] Couturier MR, Slechta ES, Goulston C, et al *Leptotrichia* bacteremia in patients receiving high-dose chemotherapy. J Clin Microbiol. 2012;50:1228–1232. 22205794 10.1128/JCM.05926-11 PMC3318514

[5] Kumagai J, Takiguchi Y, Shono K, et al Acute myelogenous leukemia with *Leptotrichia trevisanii* bacteremia. Intern Med. 2013;52:2573–2576. 24240799 10.2169/internalmedicine.52.9580

[6] Woo PCY, Wong SSY, Teng JLL, et al. *Leptotrichia hongkongensis* sp. nov., a novel *Leptotrichia* species with the oral cavity as its natural reservoir. J Zhejiang Univ Sci B. 2010;11:391–401. 20506569 10.1631/jzus.B1000056 PMC2880351

[7] Muttaiyah S, Paviour S, Buckwell L, et al Anaerobic bacteraemia in patients admitted to Auckland City Hospital: its clinical significance. N Z Med J. 2007;120:U2809. 18264176

[8] Sassone L, Fidel R, Figueiredo L, et al Evaluation of the microbiota of primary endodontic infections using checkerboard DNA-DNA hybridization. Oral Microbiol Immunol. 2007;22:390–397. 17949342 10.1111/j.1399-302X.2007.00376.x

[9] Schrimsher JM, McGuirk JP, Hinthorn DR *Leptotrichia trevisanii* sepsis after bone marrow transplantation. Emerg Infect Dis. 2013;19:1690–1691. 24047561 10.3201/eid1910.121048 PMC3810729

[10] Higurashi Y, Tatsuno K, Fujimoto F, et al Two cases of bacteremia caused by *Leptotrichia trevisanii* in patients with febrile neutropenia. J Infect Chemother. 2013;19:1181–1184. 23584842 10.1007/s10156-013-0596-7

[11] Lim YK, Kweon OJ, Kim HR, et al *Leptotrichia goodfellowii* infection: case report and literature review. Ann Clin Lab Sci. 2016;46:83–86. 26927348

[12] Matias WR, Bourque DL, Niwano T, et al Subacute bacterial endocarditis with *Leptotrichia goodfellowii* in a patient with a valvular allograft: a case report and review of the literature. Case Rep Infect Dis. 2016;2016:3051212. 27895947 10.1155/2016/3051212 PMC5118523

[13] Sabater Cabrera C, Fernández Blázquez A, García Carús E Bacteremia due to *Leptotrichia trevisanii* after an allogeneic bone marrow transplant. Enferm Infect Microbiol Clin. 2016 DOI:10.1016/j.eimc.2016.09.010 pii: S0213-005X(16)30315-9 Spanish. 27876189

[14] Cooreman S, Schuermans C, Van Schaeren J, et al Bacteraemia caused by *Leptotrichia trevisanii* in a neutropenic patient. Anaerobe. 2011;17:1–3. 21184838 10.1016/j.anaerobe.2010.12.002

[15] Baracaldo R, Bourbeau P Photo quiz: an 80-year-old female with acute myeloid leukemia and induction-associated neutropenic fever. J Clin Microbiol. 2013;51:389, 737. 23355723 10.1128/JCM.01154-12 PMC3553875

[16] Von Rosenvinge EC, Song Y, White JR, et al Immune status, antibiotic medication and pH are associated with changes in the stomach fluid microbiota. ISME J. 2013;7:1354–1366. 23466701 10.1038/ismej.2013.33 PMC3695299

[17] Martın-Gutierrez G, Rodrıguez N, Lepe JA, et al Rapid identification of a *Leptotrichia trevisanii* catheter-related bloodstream infection using matrix-assisted laser desorption/ionization time-of-flight mass spectrometry. JMM Case Reports. 2015;1–4. DOI:10.1099/jmmcr.0.000036

[18] Torres PJ, Fletcher EM, Gibbons SM, et al Characterization of the salivary microbiome in patients with pancreatic cancer. Peer J. 2015;3:e1373. 26587342 10.7717/peerj.1373 PMC4647550

[19] Guerrero-Preston R, Godoy-Vitorino F, Jedlicka A, et al 16S rRNA amplicon sequencing identifies microbiota associated with oral cancer, human papilloma virus infection and surgical treatment. Oncotarget. 2016;7:51320–51334. 27259999 10.18632/oncotarget.9710 PMC5239478

[20] Scher JU, Ubeda C, Equinda M, et al Periodontal disease and the oral microbiota in new-onset rheumatoid arthritis. Arthritis Rheum. 2012;64:3083–3094. 22576262 10.1002/art.34539 PMC3428472

[21] Boot R, Van De Berg L, Reubsaet FAG, et al. Positive *Streptobacillus moniliformis* PCR in guinea pigs likely due to *Leptotrichia* spp. Vet Microbiol. 2008;128:395–399. 18023543 10.1016/j.vetmic.2007.10.007

[22] Blairon L, De Gheldre Y, Delaere B, et al A 62-month retrospective epidemiological survey of anaerobic bacteraemia in a university hospital. Clin Microbiol Infect. 2006;12:527–532. 16700700 10.1111/j.1469-0691.2006.01407.x

[23] Preza D, Olsen I, Aas JA, et al Bacterial profiles of root caries in elderly patients. J Clin Microbiol. 2008;46:2015–2021. 18385433 10.1128/JCM.02411-07 PMC2446847

[24] Kawanami T, Fukuda K, Yatera K, et al Severe pneumonia with *Leptotrichia* sp. detected predominantly in bronchoalveolar lavage fluid by use of 16S rRNA gene sequencing analysis. J Clin Microbiol. 2009;47:496–498. 19052180 10.1128/JCM.01429-08 PMC2643685

[25] Ling Z, Kong J, Jia P, et al Analysis of oral microbiota in children with dental caries by PCR-DGGE and barcoded pyrosequencing. Microb Ecol. 2010;60:677–690. 20614117 10.1007/s00248-010-9712-8

[26] Jiang W, Jiang Y, Li C, et al Investigation of supragingival plaque microbiota in different caries status of Chinese preschool children by denaturing gradient gel electrophoresis. Microb Ecol. 2011;61:342–352. 20927511 10.1007/s00248-010-9753-z

[27] Huang S, Yang F, Zeng X, et al Preliminary characterization of the oral microbiota of Chinese adults with and without gingivitis. BMC Oral Health. 2011;11:33. 22152152 10.1186/1472-6831-11-33 PMC3254127

[28] Ahn J, Yang L, Paster BJ, et al Oral microbiome profiles: 16S rRNA pyrosequencing and microarray assay comparison. PLoS One. 2011;6:e22788. 21829515 10.1371/journal.pone.0022788 PMC3146496

[29] Cephas KD, Kim J, Mathai RA, et al Comparative analysis of salivary bacterial microbiome diversity in edentulous infants and their mothers or primary care givers using pyrosequencing. PLoS One. 2011;6:e23503. 21853142 10.1371/journal.pone.0023503 PMC3154475

[30] Pépin J, Deslandes S, Giroux G, et al The complex vaginal flora of West African women with bacterial vaginosis. PLoS One. 2011;6:e25082. 21949860 10.1371/journal.pone.0025082 PMC3176826

[31] Verhulst NO, Qiu YT, Beijleveld H, et al Composition of human skin microbiota affects attractiveness to malaria mosquitoes. PLoS One. 2011;6:e28991. 22216154 10.1371/journal.pone.0028991 PMC3247224

[32] Fethers K, Twin J, Fairley CK, et al Bacterial vaginosis (BV) candidate bacteria: associations with BV and behavioural practices in sexually-experienced and inexperienced women. PLoS One. 2012;7:e30633. 22363457 10.1371/journal.pone.0030633 PMC3281856

[33] Lo TS A cavitary pneumonia caused by *Leptotrichia* species in an immunocompetent patient. Infect Dis Rep. 2012;4:e24. 24470931 10.4081/idr.2012.e24 PMC3892657

[34] Zhu X, Wang S, Gu Y, et al Possible variation of the human oral bacterial community after wearing removable partial dentures by DGGE. World J Microbiol Biotechnol. 2012;28:2229–2236. 22806046 10.1007/s11274-012-1030-5

[35] Kumar PS, Mason MR, Brooker MR, et al Pyrosequencing reveals unique microbial signatures associated with healthy and failing dental implants. J Clin Periodontol. 2012;39:425–433. 22417294 10.1111/j.1600-051X.2012.01856.x PMC3323747

[36] Cabrera-Rubio R, Collado MC, Laitinen K, et al The human milk microbiome changes over lactation and is shaped by maternal weight and mode of delivery. Am J Clin Nutr. 2012;96:544–551. 22836031 10.3945/ajcn.112.037382

[37] Wolff D, Frese C, Maier-Kraus T, et al Bacterial biofilm composition in caries and caries-free subjects. Caries Res. 2013;47:69–77. 23147531 10.1159/000344022

[38] Ling Z, Liu X, Wang Y, et al Pyrosequencing analysis of the salivary microbiota of healthy Chinese children and adults. Microb Ecol. 2013;65:487–495. 22968328 10.1007/s00248-012-0123-x

[39] Xiang W, Li K, Liu S, et al Microbial succession in the traditional Chinese Luzhou-flavor liquor fermentation process as evaluated by SSU rRNA profiles. World J Microbiol Biotechnol. 2013;29:559–567. 23180546 10.1007/s11274-012-1210-3

[40] Warren RL, Freeman DJ, Pleasance S, et al Co-occurrence of anaerobic bacteria in colorectal carcinomas. Microbiome. 2013;1:16. 24450771 10.1186/2049-2618-1-16 PMC3971631

[41] Cheung MK, Lam WY, Fung WYW Sputum microbiota in tuberculosis as revealed by 16S rRNA pyrosequencing. PLoS One. 2013;8:e54574. 23365674 10.1371/journal.pone.0054574 PMC3554703

[42] Yang F, Huang S, He T, et al Microbial basis of oral malodor development in humans. J Dent Res. 2013;92:1106–1112. 24101743 10.1177/0022034513507065

[43] Wu J, Liu W, He L, et al Sputum microbiota associated with new, recurrent and treatment failure tuberculosis. PLoS One. 2013;8:e83445. 24349510 10.1371/journal.pone.0083445 PMC3862690

[44] Belstrøm D, Fiehn N-E, Nielsen CH, et al Altered bacterial profiles in saliva from adults with caries lesions: a case-cohort study. Caries Res. 2014;48:368–375. 24643218 10.1159/000357502

[45] Xu H, Hao W, Zhou Q, et al Plaque bacterial microbiome diversity in children younger than 30 months with or without caries prior to eruption of second primary molars. PLoS One. 2014;9:e89269. 24586647 10.1371/journal.pone.0089269 PMC3938432

[46] Li Y, Saxena D, Chen Z, et al HIV infection and microbial diversity in saliva. J Clin Microbiol. 2014;52:1400–1411. 24523469 10.1128/JCM.02954-13 PMC3993673

[47] Fernandez Y Mostajo M, Van Der Reijden WA, Buijs MJ, et al Effect of an oxygenating agent on oral bacteria *in vitro* and on dental plaque composition in healthy young adults. Front Cell Infect Microbiol. 2014;4:95. 25101249 10.3389/fcimb.2014.00095 PMC4107829

[48] Langfeldt D, Neulinger SC, Stiesch M, et al Health- and disease-associated species clusters in complex natural biofilms determine the innate immune response in oral epithelial cells during biofilm maturation. FEMS Microbiol Lett. 2014;360:137–143. 25212593 10.1111/1574-6968.12596

[49] Lif Holgerson P, Öhman C, Rönnlund A, et al Maturation of oral microbiota in children with or without dental caries. PLoS One. 2015;10:e0128534. 26020247 10.1371/journal.pone.0128534 PMC4447273

[50] Su J, Liu H-Y, Tan X-L Sputum bacterial and fungal dynamics during exacerbations of severe COPD. PLoS One. 2015;10:e0130736. 26147303 10.1371/journal.pone.0130736 PMC4493005

[51] Card RM, Mafura M, Hunt T, et al Impact of ciprofloxacin and clindamycin administration on Gram-negative bacteria isolated from healthy volunteers and characterization of the resistance genes they harbor. Antimicrob Agents Chemother. 2015;59:4410–4416. 25987611 10.1128/AAC.00068-15 PMC4505225

[52] Arvikar S, Hasturk H, Nguyen D, et al Elevated subgingival levels of periodontal pathogens in rheumatoid arthritis patients, particularly *Leptotrichia* species in new-onset disease. Abstract Number: 2721 2015 ACR/ARHP Annual Meeting; 2015 9 29 Available from: http://acrabstracts.org/abstract/elevated-subgingival-levels-of-periodontal-pathogens-in-rheumatoid-arthritis-patients-particularly-leptotrichia-species-in-new-onset-disease/

[53] Zhang M, Chen Y, Xie L, et al Pyrosequencing of plaque microflora in twin children with discordant caries phenotypes. PLoS One. 2015;10:e0141310. 26524687 10.1371/journal.pone.0141310 PMC4629883

[54] Liu S, Zhao L, Zhai Z, et al Porcine epidemic diarrhea virus infection induced the unbalance of gut microbiota in piglets. Curr Microbiol. 2015;71:643–649. 26319658 10.1007/s00284-015-0895-6

[55] Dareng EO, Ma B, Famooto AO, et al Prevalent high-risk HPV infection and vaginal microbiota in Nigerian women. Epidemiol Infect. 2016;144:123–137. 26062721 10.1017/S0950268815000965 PMC4659743

[56] Johansson I, Witkowska E, Kaveh B, et al The microbiome in populations with a low and high prevalence of caries. J Dent Res. 2016;95:80–86. 26442950 10.1177/0022034515609554 PMC4700664

[57] Wu J, Peters BA, Dominianni C, et al Cigarette smoking and the oral microbiome in a large study of American adults. ISME J. 2016;10:2435–2446. 27015003 10.1038/ismej.2016.37 PMC5030690

[58] Liu H, Guo X, Gooneratne R, et al The gut microbiome and degradation enzyme activity of wild freshwater fishes influenced by their trophic levels. Sci Rep. 2016;6:24340. 27072196 10.1038/srep24340 PMC4829839

[59] Huang S, Li Z, He T, et al Microbiota-based signature of gingivitis treatments: a randomized study. Sci Rep. 2016;6:24705. 27094556 10.1038/srep24705 PMC4837389

[60] Rashid M-U, Rosenborg S, Panagiotidis G, et al Ecological effect of solithromycin on normal human oropharyngeal and intestinal microbiota. Antimicrob Agents Chemother. 2016;60:4244–4251. pii: AAC.00461-16. 27139483 10.1128/AAC.00461-16 PMC4914664

[61] Saltykova IV, Petrov VA, Logacheva MD, et al Gallstone disease and infection with *Opisthorchis felineus* . PLoS Negl Trop Dis. 2016;10:e0004809. 27447938 10.1371/journal.pntd.0004809 PMC4957795

[62] Xiao C, Ran S, Huang Z, et al Bacterial diversity and community structure of supragingival plaques in adults with dental health or caries revealed by 16S pyrosequencing. Front Microbiol. 2016;7:1145. 27499752 10.3389/fmicb.2016.01145 PMC4956651

[63] Li Y, Zou CG, Fu Y, et al Oral microbial community typing of caries and pigment in primary dentition. BMC Genomics. 2016;17:558. 27495902 10.1186/s12864-016-2891-z PMC4974685

[64] Coit P, Mumcu G, Ture-Ozdemir F, et al Sequencing of 16S rRNA reveals a distinct salivary microbiome signature in Behçet’s disease. Clin Immunol. 2016;169:28–35. 27283393 10.1016/j.clim.2016.06.002

[65] Ren W, Zhang Q, Liu X, et al Supragingival plaque microbial community analysis of children with halitosis. J Microbiol Biotechnol. 2016;26:2141–2147. 27666996 10.4014/jmb.1605.05012

[66] Fan X, Alekseyenko AV, Wu J, et al Human oral microbiome and prospective risk for pancreatic cancer: a population-based nested case-control study. Gut. 2016 pii: gutjnl-2016-312580 DOI:10.1136/gutjnl-2016-312580. PMC5607064 27742762

[67] Hu X-Y, Yao Y-F, Cui B-M, et al. [Analysis of causes and whole microbial structure in a case of rampant caries]. Nan Fang Yi Ke Da Xue Xue Bao. 2016;36:1328–1333. Chinese. 27777193

[68] Han CS, Martin MA, Dichosa AEK, et al. Salivary microbiomes of indigenous Tsimane mothers and infants are distinct despite frequent premastication. Peer J. 2016;4:e2660. 27833819 10.7717/peerj.2660 PMC5101600

[69] Jiang S, Gao X, Jin L, et al Salivary microbiome diversity in caries-free and caries-affected children. Int J Mol Sci. 2016;17:1978. 10.3390/ijms17121978 PMC5187778 27898021

[70] Ruviére DB, Leonardo MR, Da Silva LAB, et al. Assessment of the microbiota in root canals of human primary teeth by checkerboard DNA-DNA hybridization. J Dent Child (Chic). 2007;74:118–123. 18477431

[71] Persson GR, Yeates J, Persson RE, et al The impact of a low frequency chlorhexidine rinsing schedule on the subgingival microbiota (the TEETH clinical trial). J Periodontol. 2007;78:1751–1758. 17760545 10.1902/jop.2007.070138

[72] Sassone LM, Fidel R, Faveri M, et al Microbiological evaluation of primary endodontic infections in teeth with and without sinus tract. Int Endod J. 2008;41:508–515. 18422583 10.1111/j.1365-2591.2008.01397.x

[73] Adriaens LM, Alessandri R, Spörri S, et al Does pregnancy have an impact on the subgingival microbiota? J Periodontol. 2009;80:72–81. 19228092 10.1902/jop.2009.080012

[74] Guiu A, Domingo D, Correa A, et al [*Leptotrichia goodfellowii* wound infection after a dog bite]. Rev Esp Quimioter. 2012;25: 220–221. Spanish. 22987270

[75] Kapferer I, Beier US, Jank S, et al Randomized controlled trial: lip piercing: the impact of material on microbiological findings. Pediatr Dent. 2013;35:e23–e28. 23635890

[76] Robles-Sikisaka R, Ly M, Boehm T, et al Association between living environment and human oral viral ecology. ISME J. 2013;7:1710–1724. 23598790 10.1038/ismej.2013.63 PMC3749502

[77] Murad CF, Sassone LM, Faveri M, et al Microbial diversity in persistent root canal infections investigated by checkerboard DNA-DNA hybridization. J Endod. 2014;40:899–906. 24935532 10.1016/j.joen.2014.02.010

[78] Smid MC, Dotters-Katz SK, Plongla R, et al *Leptotrichia buccalis*: a novel cause of chorioamnionitis. Infect Dis Rep. 2015;7:5801. 26294950 10.4081/idr.2015.5801 PMC4508535

[79] Renvert S, Widén C, Persson RG Cytokine and microbial profiles in relation to the clinical outcome following treatment of peri-implantitis. Clin Oral Implants Res. 2016 DOI:10.1111/clr.12927 27422156

[80] Yang I, Woltemate S, Piazuelo MB, et al Different gastric microbiota compositions in two human populations with high and low gastric cancer risk in Colombia. Sci Rep. 2016;6:18594. 26729566 10.1038/srep18594 PMC4700446

[81] Ren W, Xun Z, Wang Z, et al Tongue coating and the salivary microbial communities vary in children with halitosis. Sci Rep. 2016;6:24481. 27080513 10.1038/srep24481 PMC4832241

[82] Takeshita T, Suzuki N, Nakano Y, et al Relationship between oral malodor and the global composition of indigenous bacterial populations in saliva. Appl Environ Microbiol. 2010;76:2806–2814. 20228112 10.1128/AEM.02304-09 PMC2863426

[83] Asikainen S, Doğan B, Turgut Z, et al Specified species in gingival crevicular fluid predict bacterial diversity. PLoS One. 2010;5:e13589. 21049043 10.1371/journal.pone.0013589 PMC2963608

[84] Luo AH, Yang DQ, Xin BC, et al Microbial profiles in saliva from children with and without caries in mixed dentition. Oral Dis. 2012;18:595–601. 22458262 10.1111/j.1601-0825.2012.01915.x

[85] Bouvet P, Grégory A, Bellon L, et al [Fetal *Leptotrichia goodfellowii* bacteremia]. Med Mal Infect. 2012;42:174–175. French. 22465058 10.1016/j.medmal.2012.02.005

[86] Morou-Bermudez E, Rodriguez S, Bello AS, et al Urease and dental plaque microbial profiles in children. PLoS One. 2015;10:e0139315. 26418220 10.1371/journal.pone.0139315 PMC4587978

[87] Cho EH, Park KS, Yang M, et al Laboratory identification of *Leptotrichia* species isolated from bacteremia patients at a single institution. Ann Lab Med. 2017;37:272–276. 28224775 10.3343/alm.2017.37.3.272 PMC5339101

[88] He Y, Gong D, Shi C, et al Dysbiosis of oral buccal mucosa microbiota in patients with oral lichen planus. Oral Dis. 2017;23:674–682. 28199766 10.1111/odi.12657

[89] Janus MM, Crielaard W, Volgenant CM, et al *Candida albicans* alters the bacterial microbiome of early *in vitro* oral biofilms. J Oral Microbiol. 2017;9:1270613. 28326152 10.1080/20002297.2016.1270613 PMC5328388

[90] Ganesan SM, Joshi V, Fellows M, et al A tale of two risks: smoking, diabetes and the subgingival microbiome. ISME J. 2017;11:2075–2089. 28534880 10.1038/ismej.2017.73 PMC5563960

[91] Mok SF, Karuthan C, Cheah YK, et al The oral microbiome community variations associated with normal, potentially malignant disorders and malignant lesions of the oral cavity. Malays J Pathol. 2017;39:1–15. 28413200

[92] Chiu C-Y, Chan Y-L, Tsai Y-S, et al. Airway microbial diversity is inversely associated with mite-sensitized rhinitis and asthma in early childhood. Sci Rep. 2017;7:1820. 28500319 10.1038/s41598-017-02067-7 PMC5431806

[93] Lopes Dos Santos Santiago G, Brusselle G, Dauwe K, et al Influence of chronic azithromycin treatment on the composition of the oropharyngeal microbial community in patients with severe asthma. BMC Microbiol. 2017;17:109. 28486933 10.1186/s12866-017-1022-6 PMC5424369

[94] Duan X-B, Wu T-X, Guo Y-C, et al. Marginal bone loss around non-submerged implants is associated with salivary microbiome during bone healing. Int J Oral Sci. 2017;9:95–103. 28621324 10.1038/ijos.2017.18 PMC5518974

[95] Krieg NR, Staley JT, Brown DR, et al Bergey’s Manual of Systematic Bacteriology In: Staley JT, Whitman WB, editors. The *Bacteroidetes, Spirochaetes, Tenericutes* (*Mollicutes), Acidobacteria, Fibrobacteres, Fusobacteria, Dictyoglomi, Gemmatiminadetes, Lentisphaeraae, Verrucomicrobia, Chlamydiae*, and *Planctomycetes*. 2nd ed. Vol. 4 New York: Springer; 2005 p. 766–769.

[96] Tee W, Midolo P, Janssen PH, et al Bacteremia due to *Leptotrichia trevisanii* sp. nov. Eur J Clin Microbiol Infect Dis. 2001;20:765–769. 11783691 10.1007/s100960100618

[97] Shah HN, Olsen I, Bernard K, et al Approaches to the study of the systematics of anaerobic, gram-negative, non-sporeforming rods: current status and perspectives. Anaerobe. 2009;15:179–194. 19695337 10.1016/j.anaerobe.2009.08.003

[98] Harwich MD Jr, Serrano MG, Fettweis JM, et al Genomic sequence analysis and characterization of *Sneathia amnii* sp. nov. BMC Genomics. 2012;13:S4. 10.1186/1471-2164-13-S8-S4 PMC3535699 23281612

[99] Ivanova N, Gronow S, Lapidus A, et al Complete genome sequence of *Leptotrichia buccalis* type strain (C-1013-b). Stand Genomic Sci. 2009;1:126–132. 21304648 10.4056/sigs.1854 PMC3035221

[100] Gupta RS, Sethi M Phylogeny and molecular signatures for the phylum Fusobacteria and its distinct subclades. Anaerobe. 2014;28:182–198. 24969840 10.1016/j.anaerobe.2014.06.007

[101] Sandle T Bacteria in the blood could trigger dozens of diseases In: Science. 2016 Available from: http://www.digitaljournal.com/tech-and-science/science/bacteria-in-the-blood-could-trigger-dozens-of-diseases/article/474337?

[102] Jang JY, Song IS, Baek KJ, et al Immunologic characteristics of human gingival fibroblasts in response to oral bacteria. J Periodontal Res. 2016 DOI:10.1111/jre.12410 27558278

[103] Decroix V, Goudjil S, Kongolo G, et al ‘*Leptotrichia amnionii*’, a newly reported cause of early onset neonatal meningitis. J Med Microbiol. 2013;62:785–788. 23378562 10.1099/jmm.0.051870-0

[104] Thompson J, Pikis A Metabolism of sugars by genetically diverse species of oral *Leptotrichia* . Mol Oral Microbiol. 2012;27:34–44. 22230464 10.1111/j.2041-1014.2011.00627.x PMC3257818

[105] Birkeland NK, Hofstad T Oligosaccharides obtained by partial hydrolysis of lipopolysaccharides from *Leptotrichia buccalis* . Scand J Dent Res. 1985;93:432–435. 3864216 10.1111/j.1600-0722.1985.tb01335.x

[106] Hofstad T, Jantzen E Fatty acids of *Leptotrichia buccalis*: taxonomic implications. J Gen Microbiol. 1982;128:151–153.

[107] Shmakov S, Abudayyeh OO, Makarova KS, et al Discovery and functional characterization of diverse class 2 CRISPR-Cas systems. Mol Cell. 2015;60:385–397. 26593719 10.1016/j.molcel.2015.10.008 PMC4660269

[108] Abudayyeh OO, Gootenberg JS, Konermann S, et al C2c2 is a single-component programmable RNA-guided RNA-targeting CRISPR effector. Science. 2016;353:aaf5573. 27256883 10.1126/science.aaf5573 PMC5127784

